# Country-specific determinants for COVID-19 case fatality rate and response strategies from a global perspective: an interpretable machine learning framework

**DOI:** 10.1186/s12963-024-00330-4

**Published:** 2024-06-03

**Authors:** Cui Zhou, Åsa M. Wheelock, Chutian Zhang, Jian Ma, Zhichao Li, Wannian Liang, Jing Gao, Lei Xu

**Affiliations:** 1 Vanke School of Public Health, Tsinghua University, Beijing, China; 2 Institute for Healthy China, Tsinghua University, Beijing, China; 3 Respiratory Medicine Unit, Department of Medicine & Centre for Molecular Medicine, Karolinska Institutet, Karolinska Institutet, Slona, 171 65 Stockholm, Sweden; 4 Key Laboratory of Land Surface Pattern and Simulation, Institute of Geographic Sciences and Natural Resources Research, Chinese Academy of Sciences, Beijing, China; 5 Department of Respiratory Medicine, University of Helsinki, Helsinki, Finland; 6 The First School of Clinical Medicine, Lanzhou University, Lanzhou, China; 7 College of Natural Resources and Environment, Northwest A&F University, Yangling, China

**Keywords:** COVID-19, Global health, Strategy, Vaccination, Case fatality rate, Pandemics, XGBoost, SHAP

## Abstract

**Background:**

There are significant geographic inequities in COVID-19 case fatality rates (CFRs), and comprehensive understanding its country-level determinants in a global perspective is necessary. This study aims to quantify the country-specific risk of COVID-19 CFR and propose tailored response strategies, including vaccination strategies, in 156 countries.

**Methods:**

Cross-temporal and cross-country variations in COVID-19 CFR was identified using extreme gradient boosting (XGBoost) including 35 factors from seven dimensions in 156 countries from 28 January, 2020 to 31 January, 2022. SHapley Additive exPlanations (SHAP) was used to further clarify the clustering of countries by the key factors driving CFR and the effect of concurrent risk factors for each country. Increases in vaccination rates was simulated to illustrate the reduction of CFR in different classes of countries.

**Findings:**

Overall COVID-19 CFRs varied across countries from 28 Jan 2020 to 31 Jan 31 2022, ranging from 68 to 6373 per 100,000 population. During the COVID-19 pandemic, the determinants of CFRs first changed from health conditions to universal health coverage, and then to a multifactorial mixed effect dominated by vaccination. In the Omicron period, countries were divided into five classes according to risk determinants. Low vaccination-driven class (70 countries) mainly distributed in sub-Saharan Africa and Latin America, and include the majority of low-income countries (95.7%) with many concurrent risk factors. Aging-driven class (26 countries) mainly distributed in high-income European countries. High disease burden-driven class (32 countries) mainly distributed in Asia and North America. Low GDP-driven class (14 countries) are scattered across continents. Simulating a 5% increase in vaccination rate resulted in CFR reductions of 31.2% and 15.0% for the low vaccination-driven class and the high disease burden-driven class, respectively, with greater CFR reductions for countries with high overall risk (SHAP value > 0.1), but only 3.1% for the ageing-driven class.

**Conclusions:**

Evidence from this study suggests that geographic inequities in COVID-19 CFR is jointly determined by key and concurrent risks, and achieving a decreasing COVID-19 CFR requires more than increasing vaccination coverage, but rather targeted intervention strategies based on country-specific risks.

**Supplementary Information:**

The online version contains supplementary material available at 10.1186/s12963-024-00330-4.

## Introduction

The severe disease burden caused by COVID-19 will continue to pose a challenge to global public health systems for the foreseeable future [1–3]. As of April 2023, the pandemic has caused more than 700 million confirmed infections and over six million deaths [4]. Vaccination programs have been widely implemented around the world, but while the surge in cases and deaths has been reduced to a certain extent, it is not yet fully controlled, and inequalities in vaccine distribution have emerged [5, 6]. Health outcomes for COVID-19, including case fatality rate (CFR), vary widely across countries and could be determined by country-specific risk factors. The determinants of cross-country variation in CFRs during a COVID-19 pandemic, in the context of multiple confounding factors, are unclear. Meanwhile, there is as yet a lack of evaluation of the benefits of vaccination across countries from a global perspective, and elucidating the extent to which countries will benefit from vaccination would provide the basis for global vaccine distribution. Therefore, understanding the risk features that affect COVID-19 CFRs is critical to guide global vaccine distribution to effectively reduce CFRs.

Notably, the cross-country variation in COVID-19 CFR differs from previous patterns of infectious disease, with even geographically contiguous countries exhibiting considerable difference in CFRs. Thus, COVID-19 CFRs are widely considered to be influenced by multidimensional factors. Previous studies have tried to explain cross-country variation in COVID-19 CFR using a variety of unidimensional factors such as population age structure[7, 8], comorbidities [9, 10], medical resources [11], environment [12], culture, and so on [13]. While these studies have found some associations, they have also ignored the important interaction effects of these factors on the risk of COVID-19 death within a single country. In addition, some studies have identified complex risk factors with relevance to a single region or time period, but their findings are difficult to generalise due to that same geographical or temporal specificity [14–16]. In addition, existing studies mostly used a linear approach to explain the effects of risk factors, thereby ignoring potential non-linear effects. Building on previous research, we recognise that COVID-19 CFRs are regulated by complex factors and that identifying potential risk factors from mixed effects at the country level will provide complementary evidence for future pandemic responses.

Fast-evolving machine learning algorithms provide better analytical capabilities for real-world health emergencies. Extreme Gradient Boosting (XGBoost) is a highly optimised gradient boosting framework based on decision trees, where the algorithm iteratively combines the predictions of multiple weak learners to generate more powerful and robust models [17]. It has been widely used in medicine, chemistry, ecology, finance and other fields. Its diverse objective functions, ability to handle missing values, inclusion of regularisation terms, and easier identification of non-linear effects make it suitable for real-world health research [18]. SHapley Additive exPlanations (SHAP) is a well-established algorithm that provides a visual interpretation of the model results [19]. It can quantify the global contribution of each factor in a machine learning model, showing the direction and magnitude of each factor's effect, as well as breaking down a prediction to show how much each factor contributes to a predicted value. This enables both identification of universal risk factors in a global perspective and precise identification of each country-specific risk and its risk intensity.

Here, our study aims to identify national heterogeneity in risk factors for COVID-19 CFRs and quantify potential risks in 156 countries through the SHAP-interpreted XGboost algorithm, providing better exploratory insights into future joint interventions for the control of CFRs.

## Method

### Overview

The overall framework of this study is as follows. Firstly, we described the global distribution and epidemiological trends in CFRs, and further evaluated multidimensional features potentially affecting the heterogeneity of CFRs, including vaccination coverage, demographic factors, disease burden, behavioural risk factors, environmental risk factors, health services, and trust levels. Then, we constructed high-performance XGboost models and applied SHAP to explain those models and identify the important features affecting CFR across countries during different periods of the pandemic. After that, we clarified the country-specific risk factors for each country and their protective and risk effects on the CFR, and grouped countries into five clusters according to key risk factors. Finally, to evaluate the benefit of increasing vaccination rate on future CFR, we further simulated the change in CFR following an increase of the vaccination rate in each country.

This study complies with the Guidelines for Accurate and Transparent Health Estimates Reporting (GATHER) recommendations (Supplementary material 2.1).

### COVID-19 CFRs

Daily confirmed infections and deaths in 156 countries over the period of 28 Jan 2020 to 31 Jan 2022 were extracted from Our World in Data (OWID) [20]. Weekly CFRs were calculated from the number of new deaths and new cases per week. As there is a time-lag between deaths and cases, determined by cross-correlation analysis to be 12 days in length, we lagged the daily new deaths by 12 days to calculate the lag-adjusted weekly CFRs; we also removed countries for which less than 12 days of data were available (Supplementary material 3.1).

### SARS-CoV-2 lineage data

SARS-CoV-2 lineage data were obtained from an integrated global SARS-CoV-2 database, the China National Center for Bioinformation (CNCB), which includes data from the Global Initiative on Sharing All Influenza Data (GISAID), NCBI GenBank, National Genomics Data Center (NGDC), National Microbiology Data Center (NMDC), and China National GeneBank (CNGB). This database also provides variants identified from these sequences [21]. For each day over the study period, we determined which variant types accounted for more than 70% of all detected sequences globally, and we classified variants that met that standard as having a worldwide dominance. We defined the period of a variant's dominance as spanning from the time when the WHO defined it as a variant of concern (VOC) to the time when the next VOC appeared in no more than 10% of countries. The COVID-19 pandemic was thus divided into four periods. including the ancestral variant dominance period (original period) from 28 January to 17 December 2020, the Alpha variant dominance period (Alpha period) from 18 December 2020 to 6 April 2021, the Delta variant dominance period (Delta period) from 11 May to 21 November 2021, and the Omicron variant dominance period (Omicron period) from 26 November 2021 to 31 January 2022.

### Vaccination data

Daily vaccination data from January 28, 2020 to January 3, 2022 were extracted from OWID and pre-processed by linear interpolation in 156 countries [22]. Vaccination status was defined according to whether the last dose had been received within six months, since the protection offered by the COVID-19 vaccine drops sharply after six months [23, 24]. Vaccination rates were further organised into two categories: the proportion of the population having completed the initial vaccination protocol within six months (fully vaccinated) and that having received a booster within six months (booster given).

### Multi-dimensional explanatory variables

To comprehensively assess the risk factors influencing COVID-19 CFR, we included 35 features in six dimensions that are known or thought to affect CFRs (Table 1): demographic characteristics, national disease burden, behavioural risk factors, environmental risk factors, level of national health services, and level of trust.

**Table 1:** The list of covariates used in analyses

Short name	Dimension	Definition	Temporal coverage	Data source	Missing	Median (IQR)
Fully vaccinated	Vaccination coverage	Proportion of the population completing the initial vaccination protocol within six months	2020–2022	Our World in Data [22]	0/156	27.3 (11.4–40.0)
Booster given		Proportion of the population that received a booster dose within six months	2020–2022	Our World in Data [22]	0/156	3.9 (0–18.6)
Aged 65 +	Demographic characteristics	Proportion of population aged 65 and over	2017	UN Population Division [45]	3/156	6.6 (3.5–14.7)
Gender ratio		Number of males born per 100 females	2020	World Development Indicators—World Bank [46]	0/156	105.1 (103.9–105.9)
Average years of schooling		Average number of years the population older than 25 participated in formal education	2017	Our World in Data [47]	2/156	9.2 (6.3–11.3)
GDP per capita		Per capita gross domestic product, a measure of a country's economic output per person	2018	World Bank [48]	4/156	13,567.3 (4796.5–28,942.6)
Lower respiratory infections	Disease burden	Age-standardised prevalence of lower respiratory infections per 100,000 population	2019	Global Burden of Disease Study 2019 [49]	0/156	130.7 (84.2–184.6)
Upper respiratory infections		Age-standardised prevalence of upper respiratory infections per 100,000 population	2019	Global Burden of Disease Study 2019 [49]	0/156	3228 (2774–3712)
Chronic obstructive pulmonary disease		Age-standardised prevalence of chronic obstructive pulmonary disease per 100,000 population	2019	Global Burden of Disease Study 2019 [49]	0/156	2054.3 (1713.3–2665.0)
Cardiovascular diseases		Age-standardised prevalence of cardiovascular diseases per 100,000 population	2019	Global Burden of Disease Study 2019 [49]	0/156	6477 (5616–7132)
Stroke		Age-standardised prevalence of stroke per 100,000 population	2019	Global Burden of Disease Study 2019 [49]	0/156	1233.6 (958.2–1411.3)
Cancers		Age-standardised prevalence of cancers per 100,000 population	2019	Global Burden of Disease Study 2019 [49]	0/156	6157 (4421–9517)
Diabetes		Age-standardised prevalence of diabetes per 100,000 population	2019	Global Burden of Disease Study 2019 [49]	0/156	5490 (4243–7292)
Chronic kidney disease		Age-standardised prevalence of chronic kidney disease per 100,000 population	2019	Global Burden of Disease Study 2019 [49]	0/156	7871 (6384–9957)
Hypertension		Prevalence of hypertension among adults aged 30–79 years	2019	Global Burden of Disease Study 2019 [49]	1/156	37.3 (31.6–42.1)
Mental disorders		Age-standardised prevalence of mental disorders per 100,000 population	2019	Global Burden of Disease Study 2019 [49]	0/156	12,651 (11,365–13,971)
Noncommunicable diseases		Age-standardised total mortality from noncommunicable diseases per 100,000 population	2019	WHO [50]	0/156	90,447 (89,596–91,749)
HIV		Age-standardised prevalence of HIV infection per 100,000 population	2019	Global Burden of Disease Study 2019 [49]	0/156	176.8 (46.4–589.7)
Tuberculosis		Age-standardised prevalence of tuberculosis per 100,000 population	2019	Global Burden of Disease Study 2019 [49]	0/156	19,036 (13,148–24,781)
Overweight	Behavioural risk factor	Prevalence of overweight among adults, BMI ≥ 25 (age-standardized estimate) (%)	2016	WHO [51]	2/156	55.6 (30.9–60.6)
Low physical activity		Disability-adjusted life years attributed to low physical activity	2019	Global Burden of Disease Study 2019 [49]	0/156	188.2 (134.2–330.3)
Smoking		Disability-adjusted life years attributed to smoking	2019	Global Burden of Disease Study 2019 [49]	0/156	2024.9 (1517.8–2721.2)
Dietary risks		Disability-adjusted life years attributed to dietary risks	2019	Global Burden of Disease Study 2019 [49]	0/156	2210.2 (1607.3–2950.3)
Trees per capita	Environmental risk factor	Number of trees per capita	2014	Mapping tree density at a global scale [52]	0/156	191.0 (52.2–695.8)
PM_2.5_		Population-weighted exposure to ambient PM_2.5_ pollution (μg/m^3^) is defined as the average level of exposure of a nation's population to concentrations of suspended particles measuring less than 2.5 microns in aerodynamic diameter, which are capable of penetrating deep into the respiratory tract and causing severe health damage	2017	Global Burden of Disease Study 2019 [49]	1/156	22.2 (14.5–36.7)
Mean temperature		Mean temperature (Celsius)	1991–2020	World Bank Climate Change Knowledge Portal [53]	0/156	22.1 (11.3–25.5)
Population density		Population density (people per sq. km of land area)	2020	World Bank [54]	1/156	81.7 (31.8–160.8)
HAQ index	Health service	Healthcare Access and Quality Index	2018	GBD 2016 Healthcare Access and Quality Collaborators [32]	8/156	66.8 (41.5–81.0)
IHR score		Average of 13 International Health Regulations core capacity scores, 1st version of the questionnaire	2019	WHO [55]	0/156	66.5 (49.8–83.0)
Hospital beds		The number of hospital beds available per 10,000 inhabitants	2017	WHO [56]	3/156	20.6 (10.0–36.0)
Expenditure		Current health expenditure per capita in US$	2019	WHO [57]	4/156	392.5 (75.2–1407.3)
Hospitals		Total density of hospitals per 100,000 population	2013	WHO [58]	49/156	1.0 (0.5–1.8)
Trust the national government	Trust	Trust in the national government of the country	2020	Wellcome Global Monitor Survey [59]	55/156	52.5 (43.0–69.5)
Trust journalists		Trust in journalists in this country	2020	Wellcome Global Monitor Survey [59]	51/156	55.2 (47.4–64.8)
Trust science		Trust in science	2020	Wellcome Global Monitor Survey [59]	50/156	80.5 (70.0–88.8)

GDP, gross domestic product; PM_2.5_: particles measuring less than 2.5 microns in aerodynamic diameter; HAQ, Healthcare Access and Quality Index; IHR, International Health Regulations core capacity; WHO, World Health Organization

### XGboost

#### Model building

To develop explanatory and predictive models, we employed XGBoost algorithm to capture the non-linear associations between COVID-19 CFRs and multiple dimensional features. XGBoost is an ensemble machine learning method based on decision trees that applies a gradient boosting framework [18]. It creates a robust, more accurate prediction model from an ensemble of weak prediction models and incorporates a penalty term for model complexity to improve performance. The objective function of the XGBoost algorithm is as follows:

$$Obj(\theta ) =L(\theta )+\Omega (\theta ) =\sum_{i}L({\widehat{y}}_{i}, {y}_{i})+\sum_{k}\Omega ({f}_{k}), {f}_{k}\in F$$

where $L$ is the training loss function. $L({\widehat{y}}_{i}, {y}_{i})$ corresponds to the training loss function for each sample, where ${y}_{i}$ indicates the true value of the $i$ sample and ${\widehat{y}}_{i}$ indicates the estimated value of the $i$ sample. $\Omega$ is regularization function that measures the model’s complexity, where $k$ is the number of trees, $F$ is the set of all possible regression trees.

#### Feature selection

We filter the main features using the Recursive Feature Elimination (RFE) algorithm, which aims to capture CFR variations while retaining as few features as possible. The RFE strategy uses all the features to train the supervised model and then evaluates the features according to their importance in the model [25]. The detailed steps include: (1) Initialisation: all features are used to train the supervised model. (2) Feature importance evaluation: based on the importance of the features in the model, the least important features are selected for elimination. (3) Model update: retrain the model using the dataset with one feature removed.(4) Determine stopping condition: check whether the stopping condition is satisfied; if not, return to step 2; if it is satisfied, go to the next step. (5) Feature selection: select features from the model with better fit. In each iteration, root mean square error (RMSE) is used to evaluate the fit of the model. The model that performs best in the feature elimination process is selected as the final model. Overall, RFE finds the best subset of features for a model by progressively eliminating unimportant features, thereby reducing the number of the features while maintaining the predictive power of the model.

#### Hyperparameter tuning

The optimal set of hyperparameter values was selected using a ten-fold cross-validation grid search. The tuned parameters consisted of learning rate (from 0.05 to 0.2 with an interval of 0.05) and the maximum depth of the tree (from 4 to 10 with an interval of 1). Since our dependent variable of interest was zero-inflated right-skewed data, the objective function was set as ‘reg:tweedie’. The training process was stopped when more training cycles failed to enhance the validation dataset's performance. The dataset was split into three parts: 60% for training, 20% for validation, and 20% for testing. R^2^ and RMSE were used to assess the model's accuracy.

#### Simulation

We predicted the change in CFR under scenarios where booster vaccination rate was increased by 5% in each country. We used the best model parameters derived from the training and validation dataset, and then held all other variables constant, and changed the booster vaccination rate for each country to predict the CFRs. The principle of increasing booster vaccination is based on each country's actual full and booster vaccination rates, so we predicted CFRs for increasing booster vaccination rates within the range of a country's booster vaccination rate not exceeding the cumulative proportion of the population fully vaccinated. This approach ensured that our predictions remained within realistic limits, which reflected the actual limitations of booster vaccination coverage.

### Model interpretation

We used the SHAP framework to rank features according to their importance and explain how features affect the CFR. SHAP is a game theoretic approach that can explain the output of the XGBoost model. It connects the optimal credit allocation with a local explanation using the classical Shapley values from game theory and their associated extensions [19]. The variability of the predictions is assigned to the available features, allowing evaluation of the contribution of each feature to each prediction point. SHAP provides valuable insights into a model's behaviour by overcoming the main drawback of inconsistency in classical global feature importance measures, minimizes the possibility of underestimating the importance of a feature with a certain attribution value, shows consistency and accuracy in its importance ordering, and interpreting the model's global behaviour while retaining local faithfulness. The overall importance of a feature was scored as the mean absolute value of all SHAP values for that feature, and we considered features scoring 0.1 or higher as important [26–28]. The association between CFR and each key feature was examined via partial dependence plots, which were adjusted for all other confounding variables.

### Statistical analysis

Continuous data are presented as a mean with standard deviation (SD) where normally distributed and as a median with the 25th and 75th percentiles where non-normally distributed. We used Spearman’s rank correlation to measure the correlation of CFR with each continuous features, such as booster vaccination rate. Differences in CFRs among four groups of countries with different income levels were tested using analysis of variance (ANOVA), and then differences between pairs of country groups were tested by post-hoc tests using the Bonferroni method.

Analyses were performed in the R 4.1.1 and Python 3.8 environments.

## Results

### Temporal and regional heterogeneity of COVID-19 CFRs

**Fig. 1:**
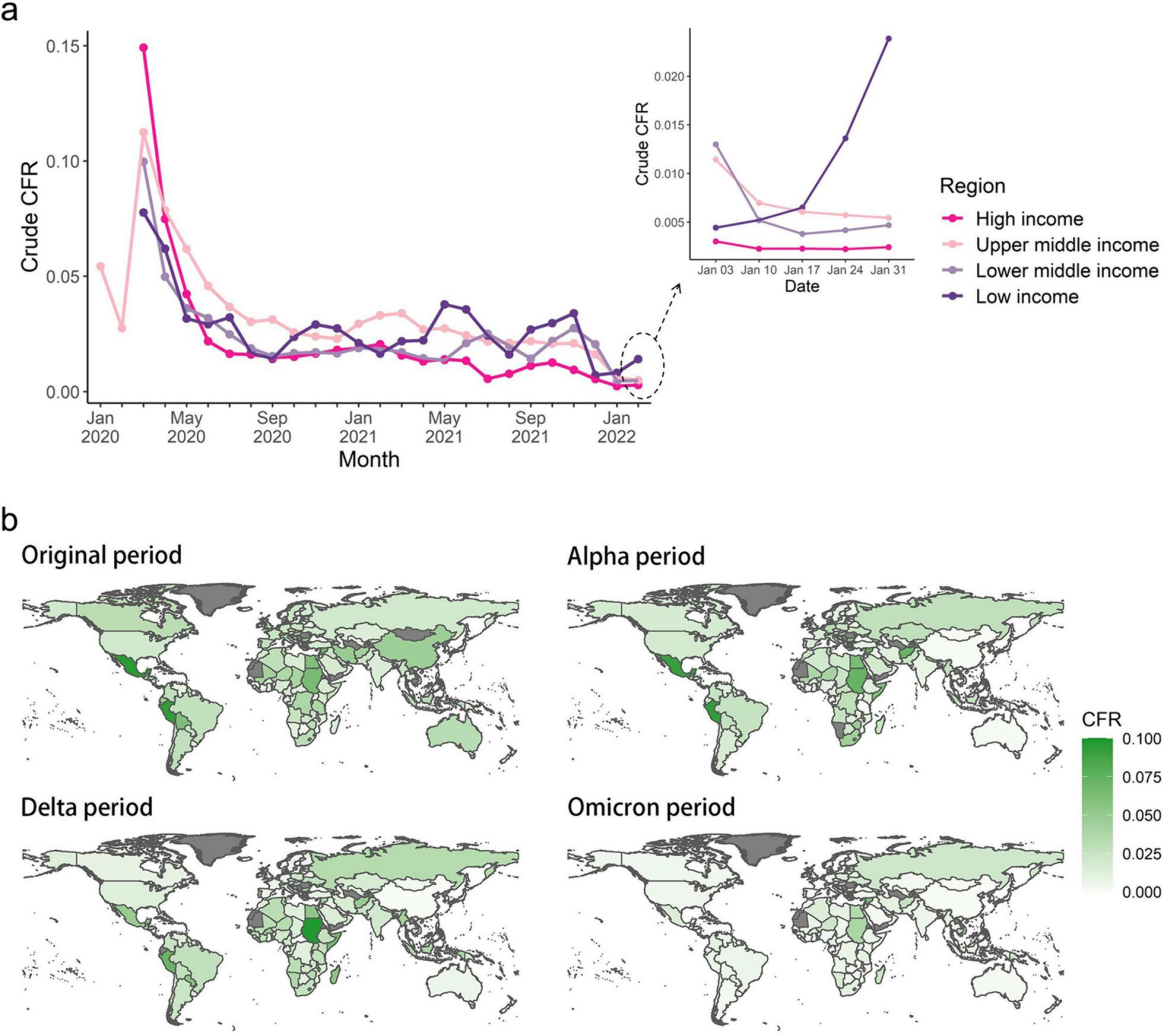
Trends in and distributions of CFR. **a** Epidemiological curves of COVID-19 CFR by WHO region from 28 January 2020 to 31 January 2022. **b** Global distribution of CFR in the original, Alpha, Delta, and Omicron periods

Overall COVID-19 CFRs varied significantly across countries, ranging from 68 per 100,000 population to 6,373 per 100,000 population. The global CFR exhibited a decreasing trend from January 2020 to January 2022, with respective values of 2.26%, 1.95%, 1.92%, and 0.74% for the original, Alpha, Delta, and Omicron periods (Fig. 1a, b). During the pandemic, CFRs gradually dropped in the high income countries after the first outbreak, while low income countries had relatively high CFRs through the end of the study period. Univariate analyses revealed significant associations with CFR for some factors such as cumulative vaccination rate, but did not satisfactorily explain the differences in CFRs across countries, for example the observation that countries with low vaccination rates always exhibit higher CFRs, but so do some countries with high vaccination rates such as Peru, Ecuador, and Mexico (Supplementary material 3.3).

### Changes in the determinants of COVID-19 CFRs over the four periods of the pandemic

**Fig. 2:**
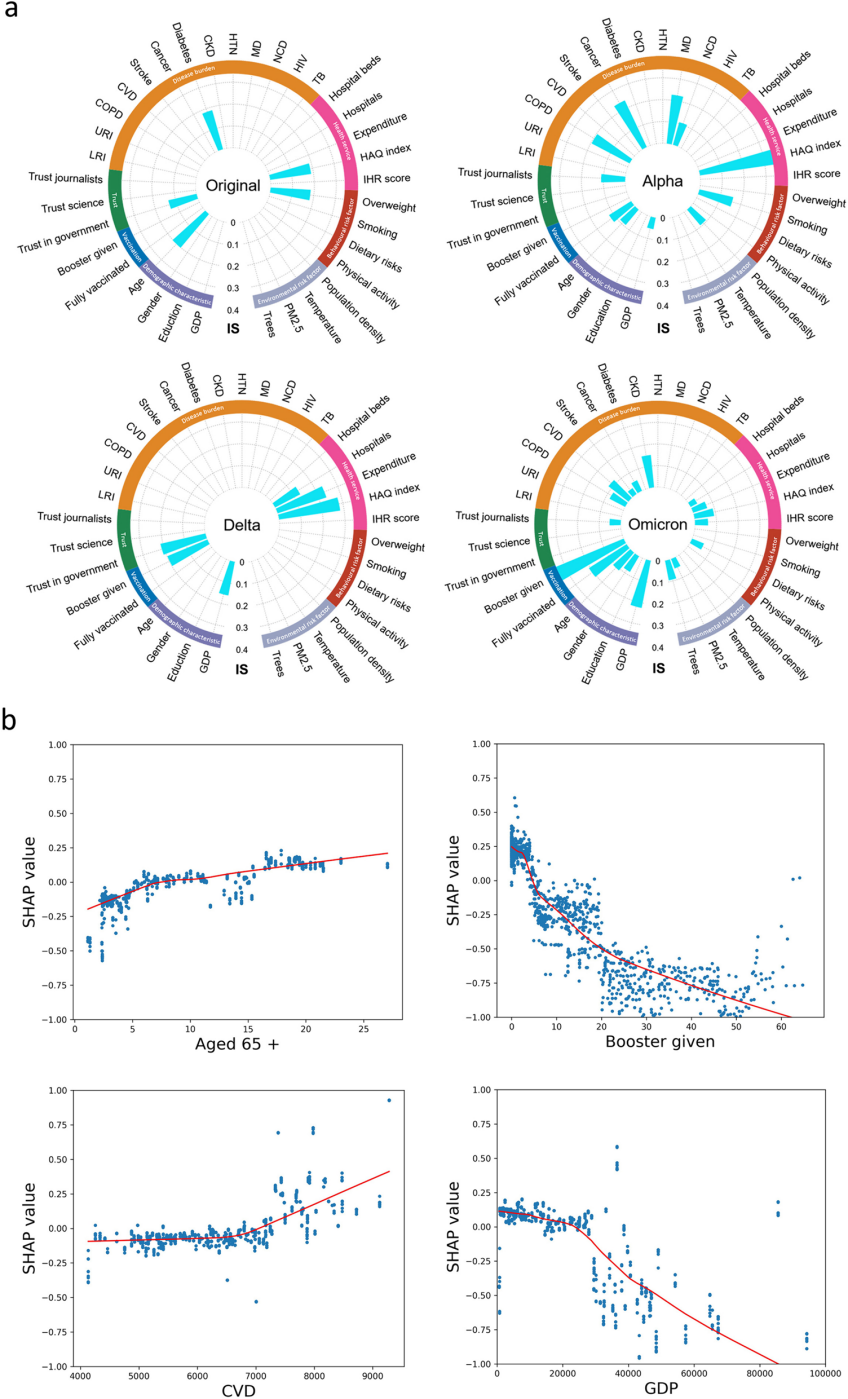
The importance of each factor affecting CFR and its effects in the original, Alpha, Delta, and Omicron periods. **a** ISs for each feature affecting CFR in each period model, obtained by taking the absolute mean of the SHAP values. The 35 features represent seven distinct dimensions: vaccination coverage, demographic factors, disease burden, behavioural risk factors, environmental risk factors, health services, and trust levels. **b** SHAP dependence plots for proportion of population aged over 65, booster vaccination rate, CVD, and GDP per capita in the XGBoost models. SHAP values above zero represent an increased risk of higher COVID-19 CFR. Abbreviations: IS, important score; LRI, lower respiratory infections; URI, upper respiratory infections; COPD, chronic obstructive pulmonary disease; CVD, cardiovascular diseases; CKD, chronic kidney disease; HTN, hypertension; MD, mental disorders; NCD, noncommunicable diseases; HIV, HIV infection; TB, tuberculosis

Most cross-country variation in CFRs in the Alpha, Delta, and Omicron periods could be well explained by the SHAP-interpreted XGboost model (R^2^: 0.76, 0.62, 0.58, respectively), but only limited interpretation was achieved for the original period (R^2^: 0.33). Important determinants of CFR and their number were found to vary across periods. From the Alpha period to the Omicron period, the important determinants first changed from health conditions to universal health coverage, and then to a multifactorial mixed effect dominated by vaccination (Fig. 2a).

The explanatory plots for each factor affecting CFR (Fig. 2b) indicate vaccination to have been an evident determinant of cross-country variation in CFRs since the Alpha period, and especially important in the Omicron period, with fully vaccinated (importance score (IS): 0.21) and booster given (IS: 0.37) status both showing a strong protective effect. From the Alpha period to the Omicron period, the protective effect of GDP on CFR gradually increased, while the importance of the HAQ index gradually decreased. In addition, ageing (IS: 0.09 and 0.11, respectively) and disease burden (IS: 0.12-0.24) were identified as important factors for increased CFR in the Alpha and Omicron periods, but not in the Delta period. A variety of disease burdens also exhibited important impacts on CFR: chronic obstructive pulmonary disease (COPD), cancers, and mental illness in the Alpha period, and cardiovascular diseases (CVD) and chronic kidney disease (CKD) in the Omicron period. Trust in government and journalists evidenced relative importance to the CFR over all four time periods (IS: 0.05-0.21). In addition, tree cover first appeared as a relatively important factor in the Omicron model.

### Country-specific determinants and concurrent risks of COVID-19 CFR

**Fig. 3:**
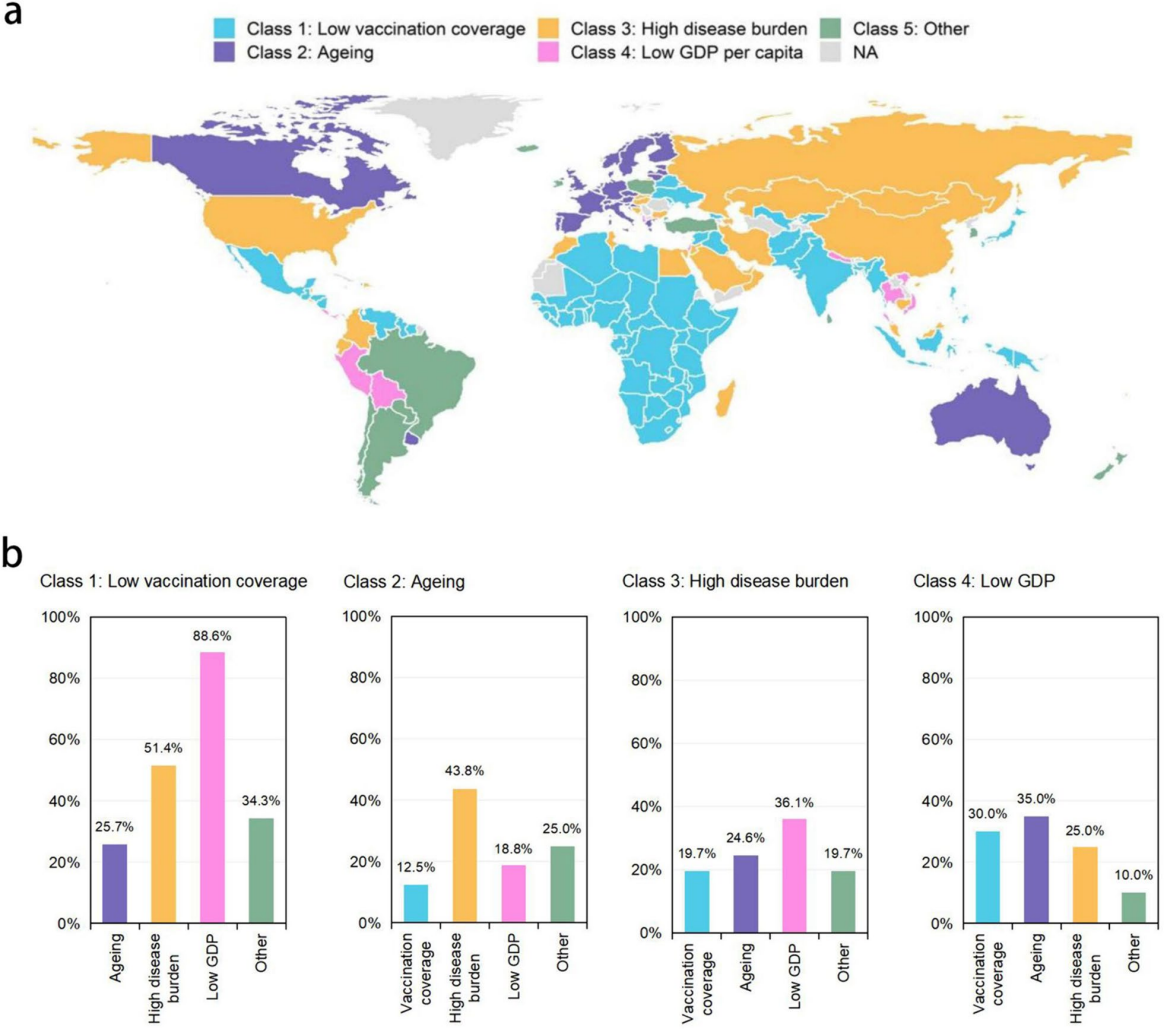
Country classification according to the most important risk factors and concurrent risks influencing COVID-19 CFR. **a** Grouping of countries into five classes based on the most important risk factors in the Omicron model. Class 1: low vaccine coverage; Class 2: ageing; Class 3: high disease burden; Class 4: low GDP; Class 5: other. **b** Percentage of countries with certain concurrent risks in each class of countries

The Omicron period model revealed that of the various determinants of CFR, the main contributors (IS > 0.1) were the population receiving booster doses and full vaccination, GDP per capita, prevalence of chronic kidney disease and cardiovascular disease, and the proportion of the population aged 65 and over. We subsequently grouped the countries into five classes based on these risks: low vaccine coverage, ageing, high disease burden, low GDP, and other (Fig. 3a). For most of the high-income countries the main risk factor is ageing (n = 26, 48.1%), in addition to 10 countries where the main risk factor is high burden of disease (18.5%), while for most of the low-income countries the main risk factor was low vaccination coverage (n = 22, 95.7%).

**Fig. 4:**
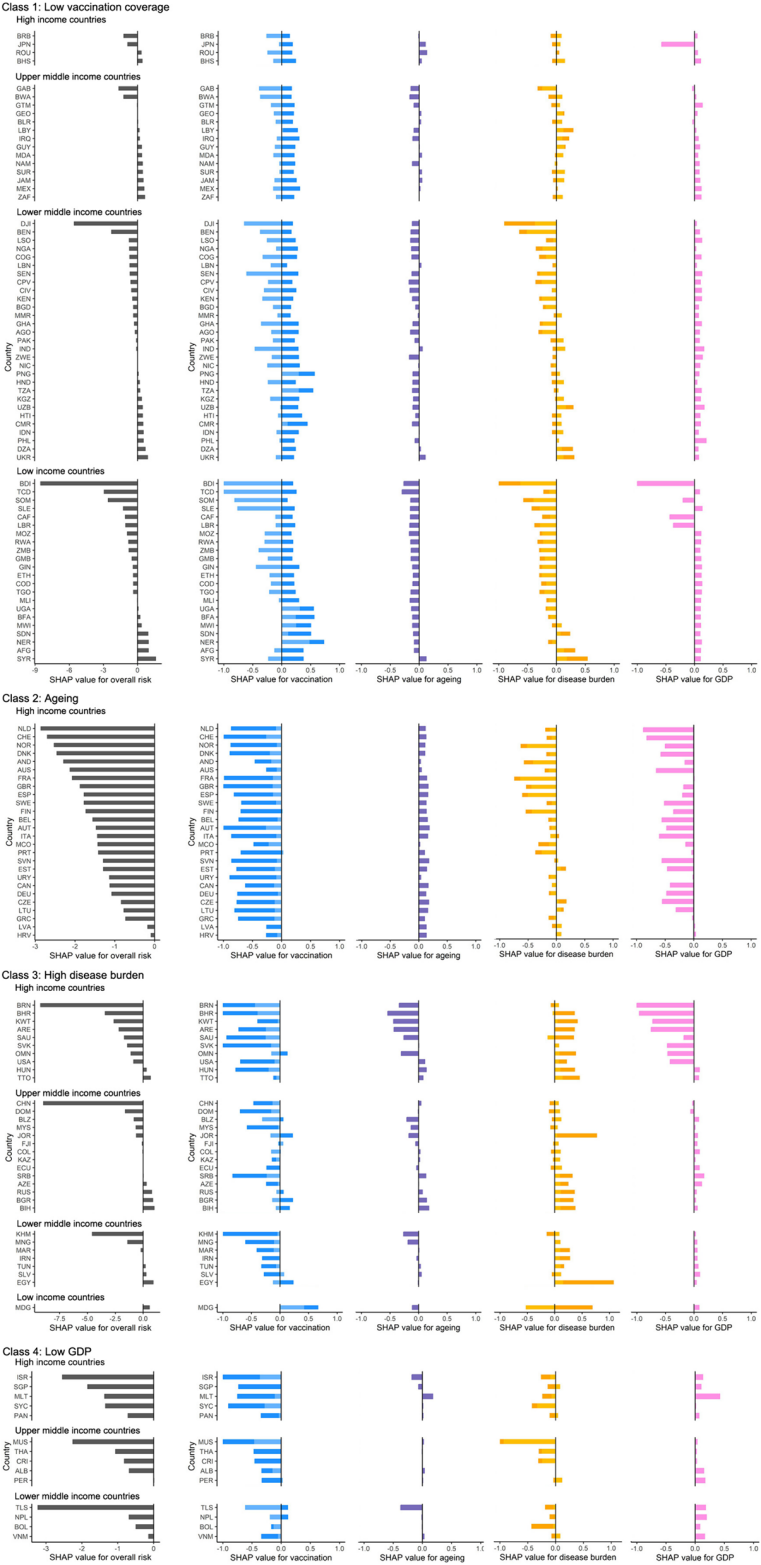
Overall risk and contributions of main risk factors to the CFR for each country in Classes 1-4. Country abbreviations use the ISO 3166 ALPHA-3 codes [44]

Figure 4 showed the total risk and the risk of each contributor for each country respectively, with SHAP values less than zero as the protective effect and greater than zero as the risk effect. For countries in Class 1 (n = 70), the main determinant of CFR was low vaccination coverage. This class was mainly comprised of countries in Africa, South East Asia and Latin America. Across all Class 1 countries only 17.1% and 0.4% of people were fully vaccinated and booster given, respectively. The highest risk due to low booster vaccination was in Sudan (SHAP value: 0.40) and due to low full vaccination was in Niger (SHAP value: 0.48) (Fig. 4). In addition, most countries in Class 1 featured multiple concurrent risk factors: 88.6% were also at risk of low GDP, and some countries (51.4%) such as Syria, Sudan, Afghanistan, and Iraq were at risk of high disease burden (Fig. 3b). For countries in Class 2, the main determinant of CFR was ageing. There are 26 countries in this class, including 23 European high-income countries such as Portugal, Germany, and Finland, as well as Canada, Australia, and Uruguay. On average, the proportion of people aged over 65 was around 19%. Countries in Class 2 had fewer concurrent risks; only seven countries, including Czechia, Estonia, and Lithuania, evidenced risk of high disease burden as a secondary determinant (Fig. 4). For countries in Class 3 (n = 32), the main determinant of CFR was high disease burden, including a high burden of CVD and CKD. Within the class, the average cardiovascular disease prevalence was 7915 per 100,000 and the average chronic kidney disease prevalence was 9,548 per 100,000. The highest risk due to CVD was in Egypt (SHAP value: 0.92), and due to CKD was in Syria (SHAP value: 0.18) (Fig. 4). Countries in Class 3 also faced more concurrent risks, with 68.8% and 46.9% being at risk of low GDP and ageing, respectively (Fig. 3b). For countries in Class 4 (n = 14), the main determinant of CFR was low GDP. This class of countries were scattered globally and characterized by fewer concurrent risks. Finally, for countries in Class 5, the main determinants of CFR comprised other factors of lesser global importance such as health expenditure, trust in journalists, and dietary risks.

### Future benefits of a 5% increase in vaccination vary by country

**Fig. 5:**
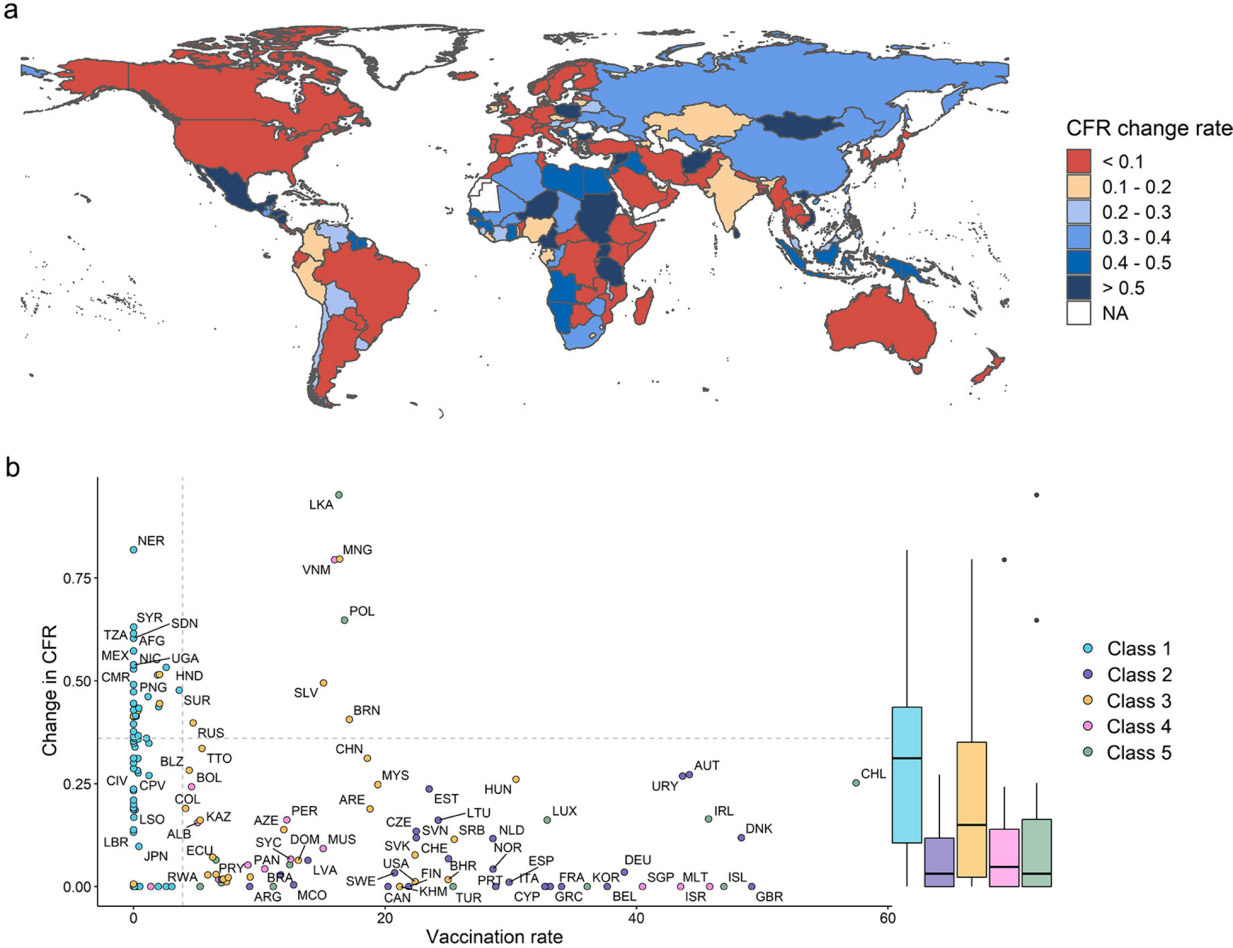
Distribution of and cross-class differences in the change in CFR after a simulated 5% increase in vaccination. **a** Global distribution of the predicted change in CFR after a 5% increase in vaccination coverage. **b** Scatter plot showing the change in CFR following increased vaccination versus current booster vaccination rate for each country. The box plot shows the distribution of change in CFR for each cluster, with boxes indicating the median and 25th and 75th percentiles

When simulating a 5% increase in vaccination, countries showed differing degrees of reduction in CFR (Fig. 5a). For countries in Class 1 and Class 3, where low vaccination rates and high disease burden constitute the main risk factors (Fig. 5b), increasing vaccination produced a greater change in CFR, with median values of 31.2% and 15.0%, respectively. Although most Class 1 countries had a significant reduction in CFRs after modelling increased vaccination rates, there were still some countries where the reduction in CFR was not significant (change rate < 0.1), e.g. Burundi, due to their lower overall risk (median SHAP value for overall risk: − 0.79) compared to other countries (median SHAP value for overall risk: 0.19). Conversely, continued increases in vaccination were of limited benefit in ageing countries (Class 2) where vaccination rates were already high, achieving a median change of 3.1%, and also in the low GDP-driven Class 4, for which the median change was 4.8%.

## Discussion

We draw three conclusions from this study. First, across the different variant dominance periods of the pandemic, the important determinants of COVID-19 CFRs changed from health conditions to universal health coverage, and then to a multifactorial mixed effect dominated by vaccination. This different weighting of factors may be due to the distinct characteristics of the respectively dominant SARS-CoV-2 strains. The higher transmissibility of the Delta variant compared to the Alpha variant may lead to its easy transmission even in healthy populations rather than a greater susceptibility in individuals with underlying disease [29]. Thus, changes in the infected population during the Delta variant period may reduce the impact of disease burden on CFR. Moreover, Delta variants result in a significant increase in the risk of hospitalisation and death in infected individuals, placing a greater burden on the healthcare system [30, 31]. Our analyses suggest that the level of the national health service is a key predictor of CFR during this period, replacing the effects of the disease burden. Adjusting investments to improve access and quality across healthcare needs will not only benefit routine care, but also improve overall health coverage in preparation for the next pandemic [32]. Furthermore, social determinants and public health interventions also affect the association between the disease burden and the CFR. Vulnerable population, such as the elderly and those with underlying diseases, are prioritised for vaccination, they have reduced CFR, which may also result in a reduction in the impact of the disease burden on the country's CFR [33]. Meanwhile, the COVID-19 pandemic took on a new pattern as a result of the emergence of the Omicron variant [34]. The immune escape characteristics of Omicron make it more contagious than earlier strains, but it also seems to be gentler, typically resulting in less severe disease [35]. In addition to the characteristics of the virus itself, patients during the Omicron period also benefited from the strong protection against severe disease and death still afforded by the COVID-19 vaccine [36]. Our study thus confirms the importance of vaccination, especially booster doses, in reducing the risk of death in Omicron pandemics. Especially in this present stage dominated by the 'Stealth' Omicron, BA.2, during which strict prevention policies are challenged by insidious transmission and the number of infections has become difficult to control, improving vaccination coverage is a cost-effective approach for reducing severe health outcomes and relieving pressure on the healthcare system.

The second major conclusion of this study is that differences in CFRs between countries are driven by effects of country-specific risk factors. Our findings highlight the noteworthy risk factors of COVID-19 death for each country at the current stage, with the most important risks being low vaccination, ageing, high disease burden, and low GDP. Based on the leading risks, we further categorized countries into four classes. Grouping countries in this way will provide joint intervention strategies for real-world policymakers and also help further a coordinated response to the pandemic that balances global and national benefits. Notably, ageing as a major risk factor was mainly found in high-income developed countries, where vaccination rates are already high and CFRs relatively low; accordingly, in addition to sustaining vaccination rates, policies in the post-COVID era may need to prioritise vulnerable populations such as older people. Similarly, countries with a high disease burden as the main risk, including some like Egypt, Madagascar, and Jordan where vaccine supply is relatively limited, would be better served by adjusting vaccine priority distribution programmes to protect the large number of vulnerable people with underlying diseases. It is also important to provide health education to these populations to enable them to accept vaccines. In another consideration, although the protective effect of vaccines has been widely demonstrated, our results suggest that in countries where low vaccination is a major risk factor, CFRs are also affected by a broad range of concurrent risks; consequently, we believe that a joint intervention would be an effective measure for reducing CFRs in this class of countries. In the short term, in addition to vaccination, a promising area for interventionists to work on is raising the level of national trust. Our findings support previous research that trust in government and science can increase risk perceptions of COVID-19 among the population, promote cooperation with outbreak prevention and control efforts, and more quickly control the number of cases and deaths [37]. Pandemics have always posed a challenge to trust between the public and the government, and maintaining and rebuilding trust during a crisis is crucial to maintaining political participation and social cohesion [38]. In the long term, behavioural factors such as smoking, obesity, diet, and nutrition, along with environmental factors such as tree cover and PM2.5, are all risk factors that can be changed through health education and policy development, and are areas in which advance preparation is needed in order to mitigate the effects of future epidemics. Regulating taxes on tobacco, tightening restrictions on smoking places, and setting a legal age for smoking would contribute to reducing the potential harm from smoking at a national level. Obesity and malnutrition are long-standing health challenges and risk factors for a range of chronic diseases, the dangers of which are already well known. However, governments also need to guide people towards healthy eating habits through policies such as requiring calorie labelling on foods and restricting the promotion of high-sugar and high-fat foods. In addition, environmental factors are of increasing concern to epidemiologists, and our research suggests that tree cover and PM_2.5_ have some impact on severe health outcomes in COVID-19. It has also been suggested that PM_2.5_ may potentially serve as a carrier for the virus [39]. Therefore, an improved environment with less air pollution would benefit both patients with COVID-19 and healthy populations.

The third major conclusion of this study is that the health benefits of continued vaccination vary between countries having different driving factors for death. On the issue of vaccine allocation, as advocated by Jeremy Bentham's Utilitarianism, a rule for society should be established that has the best outcome for the greatest amount of people in society, in the sense that a cost-effective vaccine allocation scheme should be developed in a global perspective that reduces the risk of death for the greatest proportion of people worldwide. The WHO has worked to this end by convening COVAX [40], a ground-breaking global collaboration aimed at accelerating the development and production of and equitable access to the COVID-19 vaccine, ensuring that every country has access to the vaccine and is able to promote vaccination to protect their whole population, starting with the most vulnerable. Progress on this project has not been smooth, with most early supplies of vaccine having been promptly purchased by wealthy countries and the supply shortages further exacerbated by vaccine nationalism, hoarding, and export bans. Even though COVAX has delivered more than 1.4 billion doses of vaccine to 142 countries, and 65.2% of the world's population has received at least one dose, only a cumulative 15.3% of people in low-income countries are included in that fraction [41]. This is insufficient to reach vulnerable populations such as health workers, the elderly, and people with chronic diseases. In times of inadequate vaccine supply, our model allows for real-time assessment of the risk of COVID-19 death in countries in need and of the health benefits of vaccination so as to guide vaccine allocation more rationally.

Our ecological studies based on country-level data provide a global perspective on the risk assessment of COVID-19 CFR. Country-level studies provide a more comprehensive understanding of the consistent impacts of risk factors across countries worlwide than more granular studies. We draw more generalisable conclusions at larger geographical scales, and identify key risk factors that are specific to each country, complementing the more granular studies within countries that together support policy decisions. Meanwhile, our studies provide insights into the allocation of health resources, such as vaccines, in a global perspective. Population-based and individual-based studies focus on different dimensions and issues that complement each other and contribute to a comprehensive understanding of disease development and control. For example, while there are a large number of individual-level studies across time periods that show that underlying disease is always a good predictor of death in patients with COVID-19 [42], consistent with the findings of other country-level studies that risk factors differ in importance across time periods for the national CFR, with the burden of disease from the underlying disease becoming less important during the Delta period [43]. This variation in risk factors between time periods supports policymakers in considering different intervention strategies at different times. While individual-level studies provide insights into direct health impacts, country-level studies better explain differences in disease outcomes between countries, providing a broader view of how macro-factors, such as healthcare policies and economic conditions, impact public health outcomes.

There are several limitations in our analysis. First, the study design is a country-level ecological analysis based on retrospective data, and care should be taken regarding ecological fallacies in the interpretation and generalisation of the results. Our findings do not explain CFR differences within countries, and targeted COVID-19 intervention strategies within countries may need to be supported by more fine-grained data. Second, our data were sourced from multiple publicly available data sources, and after comparing them we selected the more credible sources and also applied outlier treatment, but the credibility of our analysis relies greatly on the quality of the data. Third, COVID-19 cases and deaths are from national self-reported data and do not consider excess deaths from COVID-19. Fourth, we considered as many country-level COVID-19-related factors as possible, but due to data limitations, we were unable to adjust for differences in vaccine type and ethnicity. Fifth, the original period model has a low R^2^ value and does not capture the variation in CFR well. As the model can only explain the features we included, there may be some unknown features that we have not been able to identify.

The cross-temporal and cross-country variation in COVID-19 CFRs illustrates the importance of conducting further research on risk assessment. Our exploratory study reminds policy makers to consider risk factors holistically and assess whether their countries can rebuild policy trust, face the challenges of vaccine hesitancy, revitalize primary healthcare, and strengthen behavioural and environmental risk management and investment in the post-COVID era. At present, consideration of COVID-19 as an endemic disease has also entered the plans of some countries; that is, SARS-CoV-2 will not be eradicated and is instead expected to persist in a less lethal pattern, placing greater demands on healthcare systems and cyclical vaccination.

## Conclusions

Evidence from this study suggests that cross-temporal and cross-country variation in COVID-19 CFR is jointly determined by key and concurrent risks. Across the different variant dominance periods of the pandemic, the important determinants of COVID-19 CFRs changed from health conditions to universal health coverage, and then to a multifactorial mixed effect dominated by vaccination. We quantified the country-specific risk of COVID-19 CFR for 156 countries along seven dimensions: vaccination coverage, demographic factors, disease burden, behavioural risk factors, environmental risk factors, health services, and trust levels, and clarify the extent to which countries will benefit from increased vaccination. The findings suggested that achieving a decreasing COVID-19 case fatality rate requires more than increasing vaccination coverage, but rather targeted intervention strategies based on country-specific risks. In countries where low vaccination coverage is a major risk factor for COVID-19 deaths, increased vaccination is more effective in reducing CFR, especially in countries with high overall risk. In countries where high disease burden and ageing are major risk factors for COVID-19 deaths, it is important to focus on protection of vulnerable populations in the short term, and on interventions targeting age structure and population health status in the long term. Some risk factors that influence CFRs, such as GDP, cannot be controlled by policymakers or changed in the short term, underlining the importance of global public health efforts to strengthen cross-border cooperation to mitigate inequities.

### Supplementary Information

Supplementary material 1. 

## Data Availability

The original contributions presented in the study are included in the method/supplementary material, further inquiries can be directed to xu_lei@mail.tsinghua.edu.cn.

## Abbreviations

COVID-19: Coronavirus disease 2019
SARS-CoV-2: Severe acute respiratory syndrome coronavirus 2
CFRs: Case fatality rates
VOC: Variant of concern
XGBoost: Extreme Gradient Boosting
LASSO: Least absolute shrinkage and selection operator
SHAP: SHapley Additive exPlanations
WHO: World Health Organization
RFE: Recursive feature elimination
RMSE: Root-mean-square error
SD: Standard deviation
IQR: Interquartile range
ANOVA: Analysis of variance
HAQ Index: Healthcare access and quality index
IHR: International Health Regulations core capacity
GDP: Gross domestic product
LRI: Lower respiratory infections
URI: Upper respiratory infections
COPD: Chronic obstructive pulmonary disease
CVD: Cardiovascular diseases
CKD: Chronic kidney disease
HTN: Hypertension
MD: Mental disorders
NCD: Noncommunicable diseases
HIV: Human immunodeficiency virus infection
TB: Tuberculosis
