# Supplementary material for: Country-specific determinants for COVID-19 case fatality rate and response strategies from a global perspective: an interpretable machine learning framework
Source: Popul Health Metr. 2024 Jun 3;22:10. doi: 10.1186/s12963-024-00330-4 (PMC11149258; doi:10.1186/s12963-024-00330-4)

**Contents**

**[Section 1: Abbreviations 2](#_Toc17459)**

**[Section 2: Additional tables 3](#_Toc14450)**

[2.1 GATHER 3](#_Toc8351)

[2.2 Results of the ANOVA test and post hoc analysis for differences in CFRs across countries in four income classes 4](#_Toc23376)

[2.3 Results of feature selection using RFE 5](#_Toc19393)

[2.4 Table of SHAP values by factor for each country 17](#_Toc27215)

[2.5 Change rate of CFR after simulating a 5% increase in vaccination for each country 24](#_Toc2742)

**[Section 3: Figures 28](#_Toc4666)**

[3.1 Cross-correlation analysis for COVID-19 deaths and cases 28](#_Toc27317)

[3.2 Correlation between covariates 29](#_Toc22690)

[3.3 Univariate correlation between CFRs and vaccination rate and HAQ index 29](#_Toc7027)

[3.4 CFRs for countries in four income levels by period 30](#_Toc5413)

[3.5 SHAP dependence plot for each covariate in four period model 30](#_Toc27090)

[3.6 Distribution and cross-Class differences in the change rate of CFR after simulating a 5%, 10%, 15%, 20% increase in vaccination. 34](#_Toc19953)

# Section 1: Abbreviations

COVID-19: coronavirus disease 2019

SARS-CoV-2: severe acute respiratory syndrome coronavirus 2

CFRs: case fatality rates

VOC: variant of concern

XGBoost: Extreme Gradient Boosting

LASSO: least absolute shrinkage and selection operator

SHAP: SHapley Additive exPlanations

WHO: World Health Organization

RFE: recursive feature elimination

RMSE: root-mean-square error

SD: standard deviation

IQR: interquartile range

ANOVA: analysis of variance

HAQ Index: Healthcare Access and Quality Index

IHR: International Health Regulations core capacity

GDP: gross domestic product

LRI: lower respiratory infections

URI: upper respiratory infections

COPD: chronic obstructive pulmonary disease

CVD: cardiovascular diseases

CKD: chronic kidney disease

HTN: hypertension

MD: mental disorders

NCD: noncommunicable diseases

HIV: HIV infection

TB: tuberculosis

# Section 2: Additional tables

## 2.1 GATHER

**
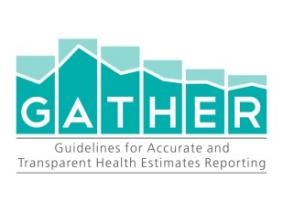
Checklist of information that should be included in new reports of global health estimates**

| **Item #** | **Checklist item** | **Reported on page #** |
| --- | --- | --- |
| **Objectives and funding** | | |
| **1** | Define the indicator(s), populations (including age, sex, and geographic entities), and time period(s) for which estimates were made. | Main Text:  Methods. |
| **2** | List the funding sources for the work. | Main Text:  Acknowledgements and  declarations. |
| **Data Inputs** | | |
| *For all data inputs from multiple sources that are synthesized as part of the study:* | | |
| **3** | Describe how the data were identified and how the data were accessed. | Main Text:  Methods and table 1. |
| **4** | Specify the inclusion and exclusion criteria. Identify all ad-hoc exclusions. | Main Text:  Methods. |
| **5** | Provide information on all included data sources and their main characteristics. For each data source used, report reference information or contact name/institution, population represented, data collection method, year(s) of data collection, sex and age range, diagnostic criteria or measurement method, and sample size, as relevant. | Main Text:  Methods and table 1. |
| **6** | Identify and describe any categories of input data that have potentially important biases (e.g., based on characteristics listed in item 5). | Main text:  Limitations. |
| *For data inputs that contribute to the analysis but were not synthesized as part of the study:* | | |
| **7** | Describe and give sources for any other data inputs. | N/A |
| *For all data inputs:* | | |
| **8** | Provide all data inputs in a file format from which data can be efficiently extracted (e.g., a spreadsheet rather than a PDF), including all relevant meta-data listed in item 5. For any data inputs that cannot be shared because of ethical or legal reasons, such as third-party ownership, provide a contact name or the name of the institution that retains the right to the data. | Main Text:  Methods and table 1. |
| **Data analysis** | | |
| **9** | Provide a conceptual overview of the data analysis method. A diagram may be helpful. | Main Text:  Methods - overview. |
| **10** | Provide a detailed description of all steps of the analysis, including mathematical formulae. This description should cover, as relevant, data cleaning, data pre-processing, data adjustments and weighting of data sources, and mathematical or statistical model(s). | Main text:  Methods. |
| **11** | Describe how candidate models were evaluated and how the final model(s) were selected. | Main text:  Introduction and Methods - XGBoost model |
| **12** | Provide the results of an evaluation of model performance, if done, as well as the results of any relevant sensitivity analysis. | Supplementary Appendix:  Section 3.6 |
| **13** | Describe methods for calculating uncertainty of the estimates. State which sources of uncertainty were, and were not, accounted for in the uncertainty analysis. | Main text:  Methods. |
| **14** | State how analytic or statistical source code used to generate estimates can be accessed. | Main text:  Methods. |
| **Results and Discussion** | | |
| **15** | Provide published estimates in a file format from which data can be efficiently extracted. | Supplementary Appendix:  Section 2.4 |
| **16** | Report a quantitative measure of the uncertainty of the estimates (e.g. uncertainty intervals). | Supplementary Appendix:  Section 3.6 |
| **17** | Interpret results in light of existing evidence. If updating a previous set of estimates, describe the reasons for changes in estimates. | N/A |
| **18** | Discuss limitations of the estimates. Include a discussion of any modelling assumptions or data limitations that affect interpretation of the estimates. | Main text:  Limitations. |

## 2.2 Results of the ANOVA test and post hoc analysis for differences in CFRs across countries in four income classes

| Pairwise comparison | Difference | Adjusted p value |
| --- | --- | --- |
| Low income-High income | 0.019 | 0 |
| Lower middle income-High income | 0.010 | 0 |
| Upper middle income-High income | 0.011 | 0 |
| Lower middle income-Low income | -0.008 | 0.036 |
| Upper middle income-Low income | -0.008 | 0.079 |
| Upper middle income-Lower middle income | 0.001 | 0.998 |

## 2.3 Results of feature selection using RFE

Alpha period model

| Number of variable | RMSE (per 1,000,000 population) | Variables |
| --- | --- | --- |
| 32 | 9573.6 | ['HAQ.index', 'Mental.disorders', 'COPD', 'Fully.vaccinated', 'Population.density', 'NCD', 'GDP.per.capita', 'Trust.journalists', 'Cancers', 'Temperature', 'Smoking', 'Trust.science', 'Gender.ratio', 'Diabetes', 'Trust.the.national.government', 'Expenditure', 'PM2.5', 'HIV', 'Aged.65.older', 'Cardiovascular.diseases', 'Trees.per.capita', 'Chronic.kidney.disease', 'IHR.score', 'Hospitals', 'Overweight', 'Hospital.beds', 'GRI', 'Upper.respiratory.infections', 'Tuberculosis', 'Lower.respiratory.infections', 'Dietary.risks', 'Booster'] |
| 11 | 9645.0 | ['HAQ.index', 'COPD', 'Mental.disorders', 'Cancers', 'Trust.journalists', 'Smoking', 'Fully.vaccinated', 'NCD', 'GDP.per.capita', 'Aged.65.older', 'Population.density'] |
| 9 | 9646.4 | ['HAQ.index', 'COPD', 'Mental.disorders', 'Cancers', 'Smoking', 'Trust.journalists', 'Fully.vaccinated', 'NCD', 'Aged.65.older'] |
| 12 | 9651.3 | ['HAQ.index', 'Mental.disorders', 'COPD', 'Trust.journalists', 'Smoking', 'Cancers', 'Fully.vaccinated', 'NCD', 'Population.density', 'Aged.65.older', 'GDP.per.capita', 'Diabetes'] |
| 34 | 9655.9 | ['HAQ.index', 'Mental.disorders', 'COPD', 'Fully.vaccinated', 'Population.density', 'Trust.journalists', 'GDP.per.capita', 'NCD', 'Smoking', 'Cancers', 'Temperature', 'Gender.ratio', 'Trust.science', 'Trust.the.national.government', 'Trees.per.capita', 'Expenditure', 'HIV', 'Diabetes', 'Chronic.kidney.disease', 'Cardiovascular.diseases', 'Aged.65.older', 'Hospitals', 'PM2.5', 'Hospital.beds', 'Overweight', 'IHR.score', 'Tuberculosis', 'GRI', 'Upper.respiratory.infections', 'Dietary.risks', 'Lower.respiratory.infections', 'Booster', 'Low.physical.activity', 'Average.years.of.schooling'] |
| 21 | 9660.4 | ['HAQ.index', 'Mental.disorders', 'COPD', 'Trust.journalists', 'Smoking', 'Fully.vaccinated', 'NCD', 'GDP.per.capita', 'Population.density', 'Cancers', 'Hospitals', 'Temperature', 'Aged.65.older', 'Diabetes', 'Chronic.kidney.disease', 'Gender.ratio', 'HIV', 'Trees.per.capita', 'Cardiovascular.diseases', 'Trust.science', 'Hospital.beds'] |
| 16 | 9683.2 | ['HAQ.index', 'Mental.disorders', 'COPD', 'Trust.journalists', 'GDP.per.capita', 'Fully.vaccinated', 'Cancers', 'Smoking', 'Population.density', 'NCD', 'Diabetes', 'Temperature', 'Aged.65.older', 'HIV', 'Gender.ratio', 'Chronic.kidney.disease'] |
| 33 | 9690.6 | ['HAQ.index', 'Mental.disorders', 'COPD', 'Fully.vaccinated', 'Population.density', 'Trust.journalists', 'NCD', 'GDP.per.capita', 'Temperature', 'Cancers', 'Smoking', 'Gender.ratio', 'Trust.science', 'Diabetes', 'Trust.the.national.government', 'Expenditure', 'PM2.5', 'HIV', 'Trees.per.capita', 'Aged.65.older', 'Chronic.kidney.disease', 'Cardiovascular.diseases', 'Hospitals', 'IHR.score', 'Overweight', 'GRI', 'Hospital.beds', 'Tuberculosis', 'Upper.respiratory.infections', 'Dietary.risks', 'Lower.respiratory.infections', 'Booster', 'Low.physical.activity'] |
| 31 | 9691.4 | ['HAQ.index', 'Mental.disorders', 'COPD', 'Fully.vaccinated', 'Population.density', 'Trust.journalists', 'Cancers', 'NCD', 'GDP.per.capita', 'Smoking', 'Gender.ratio', 'Temperature', 'Cardiovascular.diseases', 'Diabetes', 'Trust.science', 'Aged.65.older', 'Trees.per.capita', 'HIV', 'Chronic.kidney.disease', 'PM2.5', 'Expenditure', 'IHR.score', 'GRI', 'Trust.the.national.government', 'Hospitals', 'Overweight', 'Hospital.beds', 'Dietary.risks', 'Lower.respiratory.infections', 'Tuberculosis', 'Upper.respiratory.infections'] |
| 25 | 9712.1 | ['HAQ.index', 'Mental.disorders', 'COPD', 'Trust.journalists', 'Fully.vaccinated', 'NCD', 'GDP.per.capita', 'Smoking', 'Population.density', 'Cancers', 'HIV', 'Trees.per.capita', 'Temperature', 'Expenditure', 'Diabetes', 'Aged.65.older', 'Gender.ratio', 'Trust.science', 'Chronic.kidney.disease', 'Cardiovascular.diseases', 'Trust.the.national.government', 'GRI', 'Hospital.beds', 'Hospitals', 'Overweight'] |
| 29 | 9717.0 | ['HAQ.index', 'Mental.disorders', 'COPD', 'Fully.vaccinated', 'Trust.journalists', 'Population.density', 'Cancers', 'NCD', 'GDP.per.capita', 'Temperature', 'Smoking', 'Trees.per.capita', 'Trust.the.national.government', 'Gender.ratio', 'HIV', 'Diabetes', 'PM2.5', 'Expenditure', 'Aged.65.older', 'Trust.science', 'Chronic.kidney.disease', 'Hospitals', 'Lower.respiratory.infections', 'IHR.score', 'GRI', 'Hospital.beds', 'Cardiovascular.diseases', 'Upper.respiratory.infections', 'Overweight'] |
| 14 | 9723.4 | ['HAQ.index', 'Mental.disorders', 'COPD', 'Trust.journalists', 'Fully.vaccinated', 'Smoking', 'Cancers', 'NCD', 'Diabetes', 'Population.density', 'GDP.per.capita', 'HIV', 'Temperature', 'Aged.65.older'] |
| 26 | 9735.5 | ['HAQ.index', 'Mental.disorders', 'COPD', 'Trust.journalists', 'Fully.vaccinated', 'Smoking', 'NCD', 'GDP.per.capita', 'Cancers', 'Population.density', 'Temperature', 'Gender.ratio', 'HIV', 'Diabetes', 'Trust.science', 'Expenditure', 'Trees.per.capita', 'Cardiovascular.diseases', 'Hospitals', 'Trust.the.national.government', 'Chronic.kidney.disease', 'Aged.65.older', 'Hospital.beds', 'Overweight', 'GRI', 'Upper.respiratory.infections'] |
| 35 | 9736.9 | ['HAQ.index', 'Mental.disorders', 'Fully.vaccinated', 'COPD', 'Population.density', 'Hospitals', 'Trust.journalists', 'Smoking', 'Cancers', 'GDP.per.capita', 'NCD', 'Temperature', 'Aged.65.older', 'Gender.ratio', 'Trees.per.capita', 'Hospital.beds', 'Trust.science', 'Chronic.kidney.disease', 'GRI', 'Diabetes', 'HIV', 'Trust.the.national.government', 'Cardiovascular.diseases', 'Overweight', 'PM2.5', 'Expenditure', 'Lower.respiratory.infections', 'IHR.score', 'Dietary.risks', 'Tuberculosis', 'Upper.respiratory.infections', 'Low.physical.activity', 'Average.years.of.schooling', 'Booster', 'Stroke'] |
| 13 | 9737.7 | ['HAQ.index', 'Mental.disorders', 'COPD', 'Trust.journalists', 'Fully.vaccinated', 'NCD', 'Cancers', 'Smoking', 'Population.density', 'GDP.per.capita', 'Aged.65.older', 'Temperature', 'Diabetes'] |
| 5 | 9742.5 | ['HAQ.index', 'COPD', 'Mental.disorders', 'Cancers', 'Smoking'] |
| 7 | 9748.6 | ['HAQ.index', 'COPD', 'Mental.disorders', 'Cancers', 'Aged.65.older', 'Smoking', 'Trust.journalists'] |
| 6 | 9749.6 | ['HAQ.index', 'Mental.disorders', 'COPD', 'Cancers', 'Smoking', 'Trust.journalists'] |
| 20 | 9760.9 | ['HAQ.index', 'COPD', 'Mental.disorders', 'Trust.journalists', 'Fully.vaccinated', 'Smoking', 'NCD', 'GDP.per.capita', 'Population.density', 'Cancers', 'Aged.65.older', 'Gender.ratio', 'Diabetes', 'Hospitals', 'Temperature', 'HIV', 'Chronic.kidney.disease', 'Trust.science', 'Cardiovascular.diseases', 'Hospital.beds'] |
| 15 | 9765.5 | ['HAQ.index', 'Mental.disorders', 'COPD', 'Trust.journalists', 'Fully.vaccinated', 'Cancers', 'NCD', 'Smoking', 'GDP.per.capita', 'Population.density', 'Aged.65.older', 'Diabetes', 'Gender.ratio', 'Temperature', 'HIV'] |
| 8 | 9769.2 | ['HAQ.index', 'COPD', 'Mental.disorders', 'Cancers', 'Smoking', 'Trust.journalists', 'Aged.65.older', 'Fully.vaccinated'] |
| 10 | 9770.0 | ['HAQ.index', 'COPD', 'Mental.disorders', 'Cancers', 'Trust.journalists', 'NCD', 'Smoking', 'Population.density', 'Fully.vaccinated', 'Aged.65.older'] |
| 24 | 9785.4 | ['HAQ.index', 'Mental.disorders', 'COPD', 'Trust.journalists', 'Fully.vaccinated', 'NCD', 'GDP.per.capita', 'Smoking', 'Cancers', 'Population.density', 'Temperature', 'Diabetes', 'Trees.per.capita', 'Gender.ratio', 'Trust.the.national.government', 'Hospital.beds', 'Trust.science', 'HIV', 'Cardiovascular.diseases', 'Aged.65.older', 'Chronic.kidney.disease', 'GRI', 'Hospitals', 'Expenditure'] |
| 17 | 9790.2 | ['HAQ.index', 'Mental.disorders', 'COPD', 'Trust.journalists', 'GDP.per.capita', 'Fully.vaccinated', 'Cancers', 'Smoking', 'NCD', 'Diabetes', 'Population.density', 'Aged.65.older', 'Temperature', 'Gender.ratio', 'Chronic.kidney.disease', 'HIV', 'Trust.science'] |
| 27 | 9791.2 | ['HAQ.index', 'Mental.disorders', 'COPD', 'Trust.journalists', 'Fully.vaccinated', 'GDP.per.capita', 'Smoking', 'NCD', 'Population.density', 'Cancers', 'Temperature', 'Gender.ratio', 'HIV', 'Diabetes', 'Trees.per.capita', 'Expenditure', 'Hospitals', 'Trust.the.national.government', 'Trust.science', 'Chronic.kidney.disease', 'Hospital.beds', 'Aged.65.older', 'Cardiovascular.diseases', 'Overweight', 'GRI', 'Upper.respiratory.infections', 'Lower.respiratory.infections'] |
| 19 | 9807.2 | ['HAQ.index', 'Mental.disorders', 'COPD', 'Trust.journalists', 'GDP.per.capita', 'Fully.vaccinated', 'NCD', 'Cancers', 'Smoking', 'Population.density', 'Diabetes', 'Temperature', 'Aged.65.older', 'Hospitals', 'Gender.ratio', 'HIV', 'Chronic.kidney.disease', 'Cardiovascular.diseases', 'Trust.science'] |
| 23 | 9832.3 | ['HAQ.index', 'COPD', 'Mental.disorders', 'Trust.journalists', 'Fully.vaccinated', 'Smoking', 'Cancers', 'NCD', 'GDP.per.capita', 'Population.density', 'Temperature', 'Gender.ratio', 'Aged.65.older', 'Trees.per.capita', 'Diabetes', 'Trust.science', 'Cardiovascular.diseases', 'HIV', 'Trust.the.national.government', 'Chronic.kidney.disease', 'Hospital.beds', 'Hospitals', 'GRI'] |
| 18 | 9869.6 | ['HAQ.index', 'Mental.disorders', 'COPD', 'Trust.journalists', 'Fully.vaccinated', 'GDP.per.capita', 'NCD', 'Cancers', 'Smoking', 'Population.density', 'Aged.65.older', 'Diabetes', 'Temperature', 'Gender.ratio', 'HIV', 'Hospitals', 'Trust.science', 'Chronic.kidney.disease'] |
| 28 | 9886.1 | ['HAQ.index', 'COPD', 'Mental.disorders', 'Fully.vaccinated', 'Trust.journalists', 'NCD', 'Population.density', 'Smoking', 'Cancers', 'GDP.per.capita', 'Temperature', 'Trees.per.capita', 'Trust.the.national.government', 'HIV', 'Trust.science', 'Diabetes', 'Expenditure', 'Cardiovascular.diseases', 'Chronic.kidney.disease', 'Overweight', 'Gender.ratio', 'Aged.65.older', 'Lower.respiratory.infections', 'GRI', 'Hospitals', 'Upper.respiratory.infections', 'IHR.score', 'Hospital.beds'] |
| 30 | 9917.0 | ['HAQ.index', 'Mental.disorders', 'COPD', 'Fully.vaccinated', 'Trust.journalists', 'Population.density', 'GDP.per.capita', 'NCD', 'Cancers', 'Temperature', 'Smoking', 'Gender.ratio', 'Trees.per.capita', 'Aged.65.older', 'Diabetes', 'Trust.the.national.government', 'HIV', 'PM2.5', 'Chronic.kidney.disease', 'Expenditure', 'Hospitals', 'Trust.science', 'Cardiovascular.diseases', 'Lower.respiratory.infections', 'Upper.respiratory.infections', 'GRI', 'Hospital.beds', 'IHR.score', 'Overweight', 'Dietary.risks'] |
| 22 | 9917.3 | ['HAQ.index', 'Mental.disorders', 'COPD', 'Trust.journalists', 'Smoking', 'Fully.vaccinated', 'Cancers', 'NCD', 'GDP.per.capita', 'Population.density', 'Aged.65.older', 'Hospitals', 'Gender.ratio', 'HIV', 'Cardiovascular.diseases', 'Trees.per.capita', 'Diabetes', 'Temperature', 'Chronic.kidney.disease', 'Trust.science', 'GRI', 'Hospital.beds'] |

Delta period model

| Number of variable | RMSE (per 1,000,000 population) | Variables |
| --- | --- | --- |
| 6 | 12296.6 | ['HAQ.index', 'Expenditure', 'GDP.per.capita', 'Trust.the.national.government', 'Booster', 'Hospitals'] |
| 5 | 12329.6 | ['HAQ.index', 'Expenditure', 'GDP.per.capita', 'Trust.the.national.government', 'Booster'] |
| 23 | 12498.7 | ['Expenditure', 'HAQ.index', 'GDP.per.capita', 'Trust.the.national.government', 'Fully.vaccinated', 'Booster', 'Gender.ratio', 'Hospitals', 'GRI', 'Chronic.kidney.disease', 'Population.density', 'Trust.science', 'Hospital.beds', 'COPD', 'Cardiovascular.diseases', 'Upper.respiratory.infections', 'Tuberculosis', 'Mental.disorders', 'HIV', 'Smoking', 'Trust.journalists', 'Aged.65.older', 'Low.physical.activity'] |
| 25 | 12520.4 | ['Expenditure', 'HAQ.index', 'GDP.per.capita', 'Fully.vaccinated', 'Trust.the.national.government', 'Booster', 'Hospitals', 'Gender.ratio', 'GRI', 'Trust.science', 'Population.density', 'Chronic.kidney.disease', 'Smoking', 'Trust.journalists', 'Mental.disorders', 'COPD', 'Hospital.beds', 'Tuberculosis', 'Cardiovascular.diseases', 'HIV', 'Diabetes', 'Upper.respiratory.infections', 'Aged.65.older', 'Low.physical.activity', 'Lower.respiratory.infections'] |
| 34 | 12566.9 | ['HAQ.index', 'Expenditure', 'GDP.per.capita', 'Booster', 'Trust.the.national.government', 'Fully.vaccinated', 'Hospitals', 'Gender.ratio', 'GRI', 'Chronic.kidney.disease', 'COPD', 'Trust.journalists', 'Trust.science', 'HIV', 'Lower.respiratory.infections', 'Upper.respiratory.infections', 'NCD', 'Hospital.beds', 'Cancers', 'Stroke', 'Mental.disorders', 'Dietary.risks', 'Tuberculosis', 'Low.physical.activity', 'Aged.65.older', 'Smoking', 'Cardiovascular.diseases', 'Population.density', 'Diabetes', 'Average.years.of.schooling', 'PM2.5', 'Hypertension', 'Temperature', 'Overweight'] |
| 31 | 12584.8 | ['HAQ.index', 'Expenditure', 'Fully.vaccinated', 'GDP.per.capita', 'Booster', 'Trust.the.national.government', 'Hospitals', 'Gender.ratio', 'GRI', 'Trust.journalists', 'COPD', 'NCD', 'Upper.respiratory.infections', 'Trust.science', 'Chronic.kidney.disease', 'Population.density', 'Diabetes', 'Lower.respiratory.infections', 'Dietary.risks', 'Tuberculosis', 'Hospital.beds', 'Mental.disorders', 'Average.years.of.schooling', 'HIV', 'PM2.5', 'Temperature', 'Cancers', 'Aged.65.older', 'Cardiovascular.diseases', 'Smoking', 'Low.physical.activity'] |
| 21 | 12597.0 | ['HAQ.index', 'Expenditure', 'GDP.per.capita', 'Fully.vaccinated', 'Booster', 'Trust.the.national.government', 'Gender.ratio', 'Hospitals', 'Population.density', 'GRI', 'Chronic.kidney.disease', 'Mental.disorders', 'Trust.journalists', 'Trust.science', 'COPD', 'Smoking', 'Cardiovascular.diseases', 'Hospital.beds', 'Aged.65.older', 'Low.physical.activity', 'HIV'] |
| 32 | 12604.7 | ['HAQ.index', 'Expenditure', 'GDP.per.capita', 'Trust.the.national.government', 'Booster', 'Fully.vaccinated', 'Hospitals', 'Gender.ratio', 'GRI', 'Chronic.kidney.disease', 'COPD', 'Trust.journalists', 'HIV', 'Trust.science', 'Lower.respiratory.infections', 'Upper.respiratory.infections', 'NCD', 'Hospital.beds', 'Cancers', 'Low.physical.activity', 'Stroke', 'Mental.disorders', 'Tuberculosis', 'Dietary.risks', 'Aged.65.older', 'Smoking', 'Cardiovascular.diseases', 'Population.density', 'Diabetes', 'Average.years.of.schooling', 'PM2.5', 'Temperature'] |
| 29 | 12610.5 | ['Expenditure', 'HAQ.index', 'GDP.per.capita', 'Fully.vaccinated', 'Booster', 'Trust.the.national.government', 'Hospitals', 'Gender.ratio', 'Trust.journalists', 'GRI', 'COPD', 'Dietary.risks', 'Lower.respiratory.infections', 'Population.density', 'Trust.science', 'Temperature', 'HIV', 'Chronic.kidney.disease', 'Tuberculosis', 'Diabetes', 'Upper.respiratory.infections', 'Low.physical.activity', 'Cardiovascular.diseases', 'Hospital.beds', 'Smoking', 'Aged.65.older', 'Average.years.of.schooling', 'Mental.disorders', 'Cancers'] |
| 18 | 12627.9 | ['HAQ.index', 'Expenditure', 'Fully.vaccinated', 'Trust.the.national.government', 'Booster', 'GDP.per.capita', 'Hospitals', 'Chronic.kidney.disease', 'Gender.ratio', 'Population.density', 'Mental.disorders', 'GRI', 'Trust.journalists', 'Cardiovascular.diseases', 'Trust.science', 'Aged.65.older', 'Hospital.beds', 'COPD'] |
| 33 | 12628.4 | ['HAQ.index', 'Expenditure', 'GDP.per.capita', 'Booster', 'Trust.the.national.government', 'Fully.vaccinated', 'Hospitals', 'Gender.ratio', 'GRI', 'Chronic.kidney.disease', 'COPD', 'Trust.journalists', 'Trust.science', 'HIV', 'Lower.respiratory.infections', 'Upper.respiratory.infections', 'NCD', 'Stroke', 'Low.physical.activity', 'Hospital.beds', 'Cancers', 'Dietary.risks', 'Mental.disorders', 'Tuberculosis', 'Aged.65.older', 'Smoking', 'Cardiovascular.diseases', 'Diabetes', 'PM2.5', 'Population.density', 'Average.years.of.schooling', 'Hypertension', 'Temperature'] |
| 30 | 12638.6 | ['HAQ.index', 'Expenditure', 'Fully.vaccinated', 'GDP.per.capita', 'Booster', 'Trust.the.national.government', 'Hospitals', 'Gender.ratio', 'NCD', 'COPD', 'GRI', 'Chronic.kidney.disease', 'Trust.journalists', 'Upper.respiratory.infections', 'Trust.science', 'Diabetes', 'Lower.respiratory.infections', 'Dietary.risks', 'Hospital.beds', 'Population.density', 'Tuberculosis', 'Aged.65.older', 'Average.years.of.schooling', 'Mental.disorders', 'HIV', 'Cancers', 'Low.physical.activity', 'Temperature', 'Smoking', 'Cardiovascular.diseases'] |
| 24 | 12667.6 | ['HAQ.index', 'Expenditure', 'GDP.per.capita', 'Trust.the.national.government', 'Booster', 'Fully.vaccinated', 'Hospitals', 'Gender.ratio', 'GRI', 'Population.density', 'Trust.science', 'Chronic.kidney.disease', 'Smoking', 'Tuberculosis', 'COPD', 'Trust.journalists', 'HIV', 'Upper.respiratory.infections', 'Hospital.beds', 'Cardiovascular.diseases', 'Mental.disorders', 'Aged.65.older', 'Lower.respiratory.infections', 'Low.physical.activity'] |
| 7 | 12689.0 | ['HAQ.index', 'GDP.per.capita', 'Trust.the.national.government', 'Expenditure', 'Booster', 'Hospitals', 'Fully.vaccinated'] |
| 20 | 12693.4 | ['HAQ.index', 'Expenditure', 'GDP.per.capita', 'Trust.the.national.government', 'Booster', 'Fully.vaccinated', 'Hospitals', 'Population.density', 'Gender.ratio', 'Mental.disorders', 'Chronic.kidney.disease', 'GRI', 'Trust.science', 'Trust.journalists', 'Cardiovascular.diseases', 'Smoking', 'COPD', 'Hospital.beds', 'HIV', 'Aged.65.older'] |
| 11 | 12708.3 | ['HAQ.index', 'Expenditure', 'GDP.per.capita', 'Trust.the.national.government', 'Fully.vaccinated', 'Booster', 'Hospitals', 'Gender.ratio', 'Population.density', 'Mental.disorders', 'Chronic.kidney.disease'] |
| 14 | 12720.6 | ['HAQ.index', 'Expenditure', 'GDP.per.capita', 'Fully.vaccinated', 'Trust.the.national.government', 'Booster', 'Hospitals', 'Gender.ratio', 'Population.density', 'Chronic.kidney.disease', 'GRI', 'Mental.disorders', 'Cardiovascular.diseases', 'COPD'] |
| 27 | 12723.4 | ['Expenditure', 'HAQ.index', 'GDP.per.capita', 'Fully.vaccinated', 'Trust.the.national.government', 'Booster', 'Hospitals', 'Gender.ratio', 'GRI', 'Chronic.kidney.disease', 'Trust.science', 'COPD', 'Dietary.risks', 'Upper.respiratory.infections', 'Smoking', 'Population.density', 'Cardiovascular.diseases', 'HIV', 'Cancers', 'Hospital.beds', 'Low.physical.activity', 'Diabetes', 'Mental.disorders', 'Aged.65.older', 'Tuberculosis', 'Trust.journalists', 'Lower.respiratory.infections'] |
| 19 | 12757.6 | ['HAQ.index', 'Expenditure', 'GDP.per.capita', 'Trust.the.national.government', 'Booster', 'Fully.vaccinated', 'Hospitals', 'Gender.ratio', 'Chronic.kidney.disease', 'Population.density', 'Mental.disorders', 'Trust.journalists', 'GRI', 'Cardiovascular.diseases', 'Aged.65.older', 'HIV', 'Trust.science', 'COPD', 'Hospital.beds'] |
| 26 | 12760.4 | ['Expenditure', 'HAQ.index', 'GDP.per.capita', 'Booster', 'Fully.vaccinated', 'Trust.the.national.government', 'Hospitals', 'Gender.ratio', 'GRI', 'COPD', 'Chronic.kidney.disease', 'Trust.science', 'Smoking', 'Dietary.risks', 'Hospital.beds', 'Upper.respiratory.infections', 'Population.density', 'Tuberculosis', 'HIV', 'Lower.respiratory.infections', 'Trust.journalists', 'Cardiovascular.diseases', 'Low.physical.activity', 'Mental.disorders', 'Aged.65.older', 'Diabetes'] |
| 28 | 12766.7 | ['HAQ.index', 'Expenditure', 'GDP.per.capita', 'Booster', 'Fully.vaccinated', 'Trust.the.national.government', 'Gender.ratio', 'Hospitals', 'GRI', 'Smoking', 'Diabetes', 'Chronic.kidney.disease', 'COPD', 'Trust.science', 'HIV', 'Upper.respiratory.infections', 'Population.density', 'Tuberculosis', 'Trust.journalists', 'Dietary.risks', 'Low.physical.activity', 'Lower.respiratory.infections', 'Hospital.beds', 'Cancers', 'Aged.65.older', 'Mental.disorders', 'Cardiovascular.diseases', 'Temperature'] |
| 8 | 12792.1 | ['HAQ.index', 'GDP.per.capita', 'Expenditure', 'Trust.the.national.government', 'Booster', 'Hospitals', 'Fully.vaccinated', 'Population.density'] |
| 10 | 12798.5 | ['HAQ.index', 'GDP.per.capita', 'Expenditure', 'Trust.the.national.government', 'Booster', 'Fully.vaccinated', 'Hospitals', 'Gender.ratio', 'Population.density', 'Mental.disorders'] |
| 13 | 12807.5 | ['HAQ.index', 'Expenditure', 'GDP.per.capita', 'Trust.the.national.government', 'Booster', 'Fully.vaccinated', 'Hospitals', 'Gender.ratio', 'Chronic.kidney.disease', 'Mental.disorders', 'Population.density', 'GRI', 'COPD'] |
| 35 | 12814.6 | ['HAQ.index', 'Expenditure', 'GDP.per.capita', 'Fully.vaccinated', 'Booster', 'Trust.the.national.government', 'Hospitals', 'Gender.ratio', 'GRI', 'Chronic.kidney.disease', 'COPD', 'Trust.science', 'Population.density', 'PM2.5', 'HIV', 'Diabetes', 'Trust.journalists', 'Hospital.beds', 'Cancers', 'Smoking', 'NCD', 'Dietary.risks', 'Lower.respiratory.infections', 'Low.physical.activity', 'Average.years.of.schooling', 'Tuberculosis', 'Upper.respiratory.infections', 'Cardiovascular.diseases', 'Temperature', 'Aged.65.older', 'Hypertension', 'Stroke', 'IHR.score', 'Mental.disorders', 'Overweight'] |
| 15 | 12844.9 | ['HAQ.index', 'Expenditure', 'GDP.per.capita', 'Fully.vaccinated', 'Trust.the.national.government', 'Booster', 'Hospitals', 'Gender.ratio', 'Population.density', 'Chronic.kidney.disease', 'GRI', 'Mental.disorders', 'COPD', 'Cardiovascular.diseases', 'Hospital.beds'] |
| 17 | 12846.6 | ['HAQ.index', 'Expenditure', 'GDP.per.capita', 'Trust.the.national.government', 'Fully.vaccinated', 'Booster', 'Hospitals', 'Population.density', 'Gender.ratio', 'Mental.disorders', 'Chronic.kidney.disease', 'Trust.journalists', 'GRI', 'Cardiovascular.diseases', 'COPD', 'Trust.science', 'Hospital.beds'] |
| 22 | 12856.7 | ['HAQ.index', 'Expenditure', 'GDP.per.capita', 'Fully.vaccinated', 'Booster', 'Trust.the.national.government', 'Hospitals', 'Gender.ratio', 'GRI', 'Chronic.kidney.disease', 'COPD', 'Smoking', 'Trust.science', 'Mental.disorders', 'Population.density', 'Upper.respiratory.infections', 'HIV', 'Cardiovascular.diseases', 'Low.physical.activity', 'Trust.journalists', 'Aged.65.older', 'Hospital.beds'] |
| 9 | 12887.8 | ['HAQ.index', 'GDP.per.capita', 'Expenditure', 'Trust.the.national.government', 'Booster', 'Fully.vaccinated', 'Hospitals', 'Mental.disorders', 'Population.density'] |
| 16 | 12907.8 | ['HAQ.index', 'Expenditure', 'GDP.per.capita', 'Trust.the.national.government', 'Fully.vaccinated', 'Booster', 'Hospitals', 'Chronic.kidney.disease', 'Gender.ratio', 'Mental.disorders', 'Population.density', 'GRI', 'Cardiovascular.diseases', 'Trust.journalists', 'COPD', 'Hospital.beds'] |
| 12 | 12951.0 | ['HAQ.index', 'Expenditure', 'Trust.the.national.government', 'GDP.per.capita', 'Booster', 'Fully.vaccinated', 'Hospitals', 'Gender.ratio', 'Population.density', 'Mental.disorders', 'Chronic.kidney.disease', 'GRI'] |

Omicron period model

| Number of variable | RMSE (per 1,000,000 population) | Variables |
| --- | --- | --- |
| 18 | 10261.3 | ['Booster', 'Expenditure', 'Fully.vaccinated', 'GDP.per.capita', 'Chronic.kidney.disease', 'Cardiovascular.diseases', 'HAQ.index', 'Trees.per.capita', 'Gender.ratio', 'Cancers', 'Aged.65.older', 'COPD', 'Trust.journalists', 'PM2.5', 'Dietary.risks', 'IHR.score', 'Stroke', 'Hospitals'] |
| 23 | 10416.7 | ['Booster', 'Expenditure', 'Fully.vaccinated', 'GDP.per.capita', 'Cardiovascular.diseases', 'Cancers', 'Trees.per.capita', 'Chronic.kidney.disease', 'Gender.ratio', 'COPD', 'Dietary.risks', 'IHR.score', 'HAQ.index', 'Trust.science', 'Hypertension', 'PM2.5', 'Hospitals', 'Trust.journalists', 'GRI', 'Stroke', 'Hospital.beds', 'Aged.65.older', 'Population.density'] |
| 31 | 10432.5 | ['Booster', 'Expenditure', 'Fully.vaccinated', 'Chronic.kidney.disease', 'Cardiovascular.diseases', 'GDP.per.capita', 'Trees.per.capita', 'Cancers', 'HAQ.index', 'COPD', 'Gender.ratio', 'Aged.65.older', 'IHR.score', 'PM2.5', 'GRI', 'Dietary.risks', 'Trust.journalists', 'Hospital.beds', 'Trust.science', 'Hypertension', 'Lower.respiratory.infections', 'Diabetes', 'Population.density', 'Stroke', 'Tuberculosis', 'Hospitals', 'HIV', 'Low.physical.activity', 'Smoking', 'Mental.disorders', 'Overweight'] |
| 34 | 10442.9 | ['Booster', 'Expenditure', 'Fully.vaccinated', 'Cardiovascular.diseases', 'GDP.per.capita', 'Chronic.kidney.disease', 'Cancers', 'Trees.per.capita', 'COPD', 'Gender.ratio', 'HAQ.index', 'Aged.65.older', 'Dietary.risks', 'PM2.5', 'IHR.score', 'Population.density', 'Trust.journalists', 'Hospitals', 'Hospital.beds', 'Trust.science', 'GRI', 'Stroke', 'Diabetes', 'HIV', 'Low.physical.activity', 'Lower.respiratory.infections', 'Overweight', 'Smoking', 'Average.years.of.schooling', 'Hypertension', 'Mental.disorders', 'Trust.the.national.government', 'Temperature', 'Tuberculosis'] |
| 22 | 10445.0 | ['Booster', 'Expenditure', 'Fully.vaccinated', 'GDP.per.capita', 'Cardiovascular.diseases', 'Cancers', 'Chronic.kidney.disease', 'Trees.per.capita', 'COPD', 'Gender.ratio', 'Aged.65.older', 'HAQ.index', 'Dietary.risks', 'Trust.journalists', 'Stroke', 'Hospitals', 'IHR.score', 'PM2.5', 'Trust.science', 'GRI', 'Hypertension', 'Hospital.beds'] |
| 33 | 10459.6 | ['Booster', 'Expenditure', 'Fully.vaccinated', 'Cardiovascular.diseases', 'GDP.per.capita', 'Chronic.kidney.disease', 'Cancers', 'Trees.per.capita', 'COPD', 'Gender.ratio', 'Dietary.risks', 'Aged.65.older', 'PM2.5', 'HAQ.index', 'IHR.score', 'GRI', 'Hospital.beds', 'Trust.journalists', 'Stroke', 'Diabetes', 'HIV', 'Population.density', 'Hospitals', 'Low.physical.activity', 'Trust.science', 'Smoking', 'Lower.respiratory.infections', 'Overweight', 'Mental.disorders', 'Temperature', 'Average.years.of.schooling', 'Tuberculosis', 'Hypertension'] |
| 32 | 10474.9 | ['Booster', 'Expenditure', 'Fully.vaccinated', 'Cardiovascular.diseases', 'GDP.per.capita', 'Chronic.kidney.disease', 'Cancers', 'Trees.per.capita', 'COPD', 'Gender.ratio', 'Aged.65.older', 'Dietary.risks', 'IHR.score', 'PM2.5', 'Trust.journalists', 'Stroke', 'HAQ.index', 'Low.physical.activity', 'Hospital.beds', 'Lower.respiratory.infections', 'Hospitals', 'Trust.science', 'GRI', 'Population.density', 'HIV', 'Overweight', 'Mental.disorders', 'Diabetes', 'Smoking', 'Hypertension', 'Temperature', 'Tuberculosis'] |
| 24 | 10488.0 | ['Booster', 'Expenditure', 'Fully.vaccinated', 'GDP.per.capita', 'Cardiovascular.diseases', 'Chronic.kidney.disease', 'Trees.per.capita', 'Cancers', 'COPD', 'Gender.ratio', 'Dietary.risks', 'Aged.65.older', 'HAQ.index', 'IHR.score', 'GRI', 'Stroke', 'Hospitals', 'Trust.science', 'PM2.5', 'Trust.journalists', 'Hospital.beds', 'Hypertension', 'Population.density', 'Lower.respiratory.infections'] |
| 19 | 10501.8 | ['Booster', 'Expenditure', 'Fully.vaccinated', 'GDP.per.capita', 'Cardiovascular.diseases', 'Chronic.kidney.disease', 'Cancers', 'Trees.per.capita', 'COPD', 'Gender.ratio', 'HAQ.index', 'Aged.65.older', 'PM2.5', 'Dietary.risks', 'Hospitals', 'Stroke', 'IHR.score', 'GRI', 'Trust.journalists'] |
| 14 | 10504.5 | ['Booster', 'Expenditure', 'Fully.vaccinated', 'Chronic.kidney.disease', 'GDP.per.capita', 'Cancers', 'Trees.per.capita', 'Cardiovascular.diseases', 'COPD', 'Gender.ratio', 'HAQ.index', 'Dietary.risks', 'Aged.65.older', 'Trust.journalists'] |
| 35 | 10514.1 | ['Booster', 'Expenditure', 'Fully.vaccinated', 'Cardiovascular.diseases', 'GDP.per.capita', 'Trees.per.capita', 'Chronic.kidney.disease', 'Cancers', 'Gender.ratio', 'COPD', 'Aged.65.older', 'HAQ.index', 'PM2.5', 'IHR.score', 'Dietary.risks', 'GRI', 'Trust.journalists', 'Hospital.beds', 'HIV', 'Stroke', 'Trust.science', 'Population.density', 'Diabetes', 'Temperature', 'Hospitals', 'Tuberculosis', 'Overweight', 'Lower.respiratory.infections', 'Smoking', 'Low.physical.activity', 'Average.years.of.schooling', 'Trust.the.national.government', 'Upper.respiratory.infections', 'Mental.disorders', 'Hypertension'] |
| 20 | 10528.3 | ['Booster', 'Expenditure', 'Fully.vaccinated', 'GDP.per.capita', 'Cardiovascular.diseases', 'Trees.per.capita', 'Cancers', 'Chronic.kidney.disease', 'Gender.ratio', 'COPD', 'Dietary.risks', 'PM2.5', 'HAQ.index', 'Aged.65.older', 'Hospitals', 'Stroke', 'GRI', 'Trust.journalists', 'IHR.score', 'Trust.science'] |
| 15 | 10532.5 | ['Booster', 'Fully.vaccinated', 'Expenditure', 'GDP.per.capita', 'Chronic.kidney.disease', 'HAQ.index', 'Cancers', 'Cardiovascular.diseases', 'Trees.per.capita', 'Dietary.risks', 'COPD', 'Aged.65.older', 'Gender.ratio', 'Trust.journalists', 'IHR.score'] |
| 17 | 10539.9 | ['Booster', 'Expenditure', 'Fully.vaccinated', 'GDP.per.capita', 'Cardiovascular.diseases', 'Chronic.kidney.disease', 'Trees.per.capita', 'Cancers', 'Dietary.risks', 'HAQ.index', 'Gender.ratio', 'COPD', 'Aged.65.older', 'Trust.journalists', 'IHR.score', 'Stroke', 'PM2.5'] |
| 29 | 10555.9 | ['Booster', 'Expenditure', 'Fully.vaccinated', 'Cardiovascular.diseases', 'Chronic.kidney.disease', 'GDP.per.capita', 'Cancers', 'Trees.per.capita', 'COPD', 'Gender.ratio', 'HAQ.index', 'PM2.5', 'Trust.journalists', 'Aged.65.older', 'IHR.score', 'Hospital.beds', 'GRI', 'Dietary.risks', 'Stroke', 'Low.physical.activity', 'Trust.science', 'Diabetes', 'HIV', 'Hospitals', 'Population.density', 'Hypertension', 'Overweight', 'Lower.respiratory.infections', 'Tuberculosis'] |
| 25 | 10581.4 | ['Booster', 'Expenditure', 'Fully.vaccinated', 'GDP.per.capita', 'Chronic.kidney.disease', 'Cardiovascular.diseases', 'Trees.per.capita', 'Cancers', 'COPD', 'Gender.ratio', 'HAQ.index', 'Aged.65.older', 'Dietary.risks', 'Trust.journalists', 'IHR.score', 'Hospital.beds', 'PM2.5', 'Stroke', 'GRI', 'Hospitals', 'Trust.science', 'Lower.respiratory.infections', 'HIV', 'Population.density', 'Hypertension'] |
| 28 | 10598.3 | ['Booster', 'Expenditure', 'Fully.vaccinated', 'Cardiovascular.diseases', 'GDP.per.capita', 'Chronic.kidney.disease', 'Cancers', 'Trees.per.capita', 'COPD', 'Gender.ratio', 'Trust.journalists', 'Aged.65.older', 'PM2.5', 'IHR.score', 'HAQ.index', 'Hospital.beds', 'Trust.science', 'Dietary.risks', 'GRI', 'Stroke', 'Low.physical.activity', 'Hospitals', 'Diabetes', 'Population.density', 'Hypertension', 'HIV', 'Lower.respiratory.infections', 'Overweight'] |
| 30 | 10598.8 | ['Booster', 'Expenditure', 'Fully.vaccinated', 'Cardiovascular.diseases', 'GDP.per.capita', 'Cancers', 'Trees.per.capita', 'HAQ.index', 'Chronic.kidney.disease', 'COPD', 'Gender.ratio', 'Aged.65.older', 'IHR.score', 'PM2.5', 'Trust.journalists', 'Trust.science', 'Dietary.risks', 'Stroke', 'HIV', 'Diabetes', 'GRI', 'Hospital.beds', 'Population.density', 'Lower.respiratory.infections', 'Hospitals', 'Hypertension', 'Low.physical.activity', 'Mental.disorders', 'Tuberculosis', 'Overweight'] |
| 21 | 10633.1 | ['Booster', 'Expenditure', 'Fully.vaccinated', 'GDP.per.capita', 'Chronic.kidney.disease', 'Trees.per.capita', 'Cancers', 'Aged.65.older', 'Cardiovascular.diseases', 'COPD', 'HAQ.index', 'Dietary.risks', 'Gender.ratio', 'IHR.score', 'Stroke', 'Trust.journalists', 'Trust.science', 'PM2.5', 'GRI', 'Hospitals', 'Hypertension'] |
| 26 | 10677.9 | ['Booster', 'Expenditure', 'Fully.vaccinated', 'GDP.per.capita', 'Cardiovascular.diseases', 'Chronic.kidney.disease', 'Cancers', 'Trees.per.capita', 'COPD', 'Gender.ratio', 'HAQ.index', 'Aged.65.older', 'Trust.journalists', 'Dietary.risks', 'IHR.score', 'Stroke', 'PM2.5', 'HIV', 'Hospital.beds', 'Hospitals', 'Lower.respiratory.infections', 'Trust.science', 'Population.density', 'GRI', 'Hypertension', 'Overweight'] |
| 16 | 10687.2 | ['Booster', 'Expenditure', 'Fully.vaccinated', 'GDP.per.capita', 'Chronic.kidney.disease', 'Cardiovascular.diseases', 'HAQ.index', 'Cancers', 'Trees.per.capita', 'Aged.65.older', 'Gender.ratio', 'COPD', 'Dietary.risks', 'IHR.score', 'Trust.journalists', 'PM2.5'] |
| 13 | 10707.0 | ['Booster', 'Expenditure', 'Fully.vaccinated', 'Chronic.kidney.disease', 'GDP.per.capita', 'Trees.per.capita', 'Cancers', 'Cardiovascular.diseases', 'HAQ.index', 'Gender.ratio', 'COPD', 'Dietary.risks', 'Trust.journalists'] |
| 27 | 10730.1 | ['Booster', 'Fully.vaccinated', 'Expenditure', 'GDP.per.capita', 'Cardiovascular.diseases', 'Chronic.kidney.disease', 'Trees.per.capita', 'Cancers', 'COPD', 'Gender.ratio', 'HAQ.index', 'Aged.65.older', 'Trust.journalists', 'PM2.5', 'Trust.science', 'Hospitals', 'Lower.respiratory.infections', 'Dietary.risks', 'IHR.score', 'Stroke', 'Population.density', 'HIV', 'Hospital.beds', 'GRI', 'Overweight', 'Hypertension', 'Low.physical.activity'] |
| 12 | 10770.7 | ['Booster', 'Expenditure', 'Fully.vaccinated', 'Chronic.kidney.disease', 'GDP.per.capita', 'Trees.per.capita', 'Cancers', 'Cardiovascular.diseases', 'HAQ.index', 'Gender.ratio', 'COPD', 'Dietary.risks'] |
| 11 | 10808.0 | ['Booster', 'Expenditure', 'Fully.vaccinated', 'Chronic.kidney.disease', 'GDP.per.capita', 'Trees.per.capita', 'Cardiovascular.diseases', 'Cancers', 'COPD', 'Gender.ratio', 'HAQ.index'] |
| 10 | 11018.7 | ['Booster', 'Expenditure', 'GDP.per.capita', 'Fully.vaccinated', 'Cardiovascular.diseases', 'Trees.per.capita', 'Chronic.kidney.disease', 'Cancers', 'HAQ.index', 'Gender.ratio'] |
| 5 | 11054.0 | ['GDP.per.capita', 'Booster', 'Expenditure', 'Chronic.kidney.disease', 'Fully.vaccinated'] |
| 7 | 11108.6 | ['Booster', 'GDP.per.capita', 'Expenditure', 'Fully.vaccinated', 'Chronic.kidney.disease', 'Cancers', 'Trees.per.capita'] |
| 6 | 11158.2 | ['Booster', 'GDP.per.capita', 'Expenditure', 'Cancers', 'Fully.vaccinated', 'Chronic.kidney.disease'] |
| 9 | 11214.8 | ['Booster', 'GDP.per.capita', 'Expenditure', 'Fully.vaccinated', 'Trees.per.capita', 'Cancers', 'Chronic.kidney.disease', 'Cardiovascular.diseases', 'Gender.ratio'] |
| 8 | 11303.2 | ['Booster', 'GDP.per.capita', 'Expenditure', 'Fully.vaccinated', 'Chronic.kidney.disease', 'Trees.per.capita', 'Cardiovascular.diseases', 'Cancers'] |

## 2.4 Table of SHAP values by factor for each country

| **Entity** | **Booster given** | **GDP per capita** | **Fully vaccinated** | **Chronic kidney disease** | **Cardiovascular diseases** | **Aged 65 older** | **Other** |
| --- | --- | --- | --- | --- | --- | --- | --- |
| **Africa** |  |  |  |  |  |  |  |
| Burkina Faso | 0.322 | 0.103 | 0.244 | -0.107 | -0.037 | -0.115 | -0.163 |
| Burundi | 0.199 | -3.017 | -2.492 | -1.737 | -0.374 | -0.265 | -0.850 |
| Central African Republic | 0.188 | -0.432 | -0.109 | -0.109 | -0.137 | -0.152 | -0.345 |
| Chad | 0.257 | 0.090 | -1.308 | -0.124 | -0.101 | -0.295 | -1.481 |
| Democratic Republic of Congo | 0.220 | 0.129 | -0.186 | -0.175 | -0.089 | -0.111 | -0.170 |
| Ethiopia | 0.214 | 0.119 | -0.208 | -0.254 | -0.044 | -0.103 | -0.118 |
| Gambia | 0.184 | 0.115 | -0.239 | -0.202 | -0.087 | -0.142 | -0.143 |
| Guinea | 0.304 | 0.124 | -0.446 | -0.248 | -0.047 | -0.115 | 0.017 |
| Liberia | 0.234 | -0.368 | -0.104 | -0.284 | -0.097 | -0.164 | -0.283 |
| Madagascar | 0.248 | 0.087 | 0.423 | -0.529 | 0.693 | -0.111 | -0.211 |
| Malawi | 0.261 | 0.109 | 0.249 | 0.092 | -0.077 | -0.114 | -0.159 |
| Mali | 0.301 | 0.108 | -0.049 | -0.127 | -0.048 | -0.159 | -0.065 |
| Mozambique | 0.172 | 0.114 | -0.293 | -0.246 | -0.041 | -0.172 | -0.461 |
| Niger | 0.254 | 0.122 | 0.480 | -0.071 | -0.071 | -0.085 | 0.355 |
| Rwanda | 0.201 | 0.110 | -0.296 | -0.229 | -0.100 | -0.148 | -0.345 |
| Sierra Leone | 0.225 | 0.135 | -0.765 | -0.288 | -0.141 | -0.153 | -0.285 |
| Sudan | 0.399 | 0.098 | 0.112 | 0.072 | 0.167 | -0.099 | 0.195 |
| Togo | 0.243 | 0.121 | -0.216 | -0.208 | -0.087 | -0.144 | -0.090 |
| Uganda | 0.243 | 0.103 | 0.314 | -0.156 | -0.028 | -0.134 | -0.246 |
| Algeria | 0.227 | 0.062 | 0.019 | 0.092 | 0.194 | 0.022 | 0.096 |
| Angola | 0.294 | 0.087 | -0.179 | -0.245 | -0.067 | -0.151 | 0.025 |
| Benin | 0.172 | 0.089 | -0.375 | -0.513 | -0.132 | -0.144 | -1.412 |
| Cameroon | 0.346 | 0.104 | 0.103 | 0.090 | -0.073 | -0.118 | 0.063 |
| Cape Verde | 0.185 | 0.104 | -0.234 | -0.255 | -0.107 | -0.176 | -0.126 |
| Congo | 0.265 | 0.116 | -0.328 | -0.179 | -0.122 | -0.129 | -0.339 |
| Cote d'Ivoire | 0.253 | 0.121 | -0.305 | -0.073 | -0.008 | -0.160 | -0.384 |
| Ghana | 0.294 | 0.122 | -0.357 | -0.237 | -0.051 | -0.109 | 0.031 |
| Kenya | 0.200 | 0.124 | -0.334 | -0.255 | -0.043 | -0.117 | -0.035 |
| Lesotho | 0.242 | 0.128 | -0.257 | -0.056 | -0.123 | -0.148 | -0.540 |
| Nigeria | 0.280 | 0.024 | -0.096 | -0.242 | -0.115 | -0.135 | -0.460 |
| Senegal | 0.287 | 0.130 | -0.611 | -0.286 | -0.049 | -0.127 | -0.026 |
| Tanzania | 0.250 | 0.120 | 0.296 | 0.036 | -0.045 | -0.117 | -0.300 |
| Zambia | 0.199 | 0.095 | -0.400 | -0.265 | -0.036 | -0.139 | -0.230 |
| Zimbabwe | 0.300 | 0.139 | -0.171 | -0.012 | -0.055 | -0.170 | -0.063 |
| Botswana | 0.171 | 0.024 | -0.373 | 0.103 | -0.137 | -0.162 | -0.867 |
| Gabon | 0.178 | -0.034 | -0.391 | -0.247 | -0.080 | -0.142 | -0.956 |
| Mauritius | -0.602 | 0.031 | -0.461 | -0.955 | -0.069 | 0.019 | -0.223 |
| Namibia | 0.234 | 0.083 | -0.040 | -0.028 | 0.017 | -0.118 | 0.315 |
| South Africa | 0.218 | 0.116 | -0.100 | 0.111 | -0.063 | 0.000 | 0.379 |
| **High income** |  |  |  |  |  |  |  |
| Seychelles | -0.625 | 0.009 | -0.283 | -0.328 | -0.096 | 0.009 | -0.037 |
| **Americas** |  |  |  |  |  |  |  |
| Belize | 0.061 | 0.082 | -0.312 | 0.122 | -0.050 | -0.206 | -0.539 |
| Bolivia | -0.041 | 0.080 | -0.129 | -0.045 | -0.388 | -0.003 | 0.025 |
| El Salvador | -0.283 | 0.099 | 0.074 | 0.119 | -0.052 | 0.047 | 0.303 |
| Haiti | 0.352 | 0.098 | -0.064 | 0.085 | -0.076 | -0.062 | 0.140 |
| Honduras | 0.244 | 0.043 | -0.240 | 0.130 | -0.081 | -0.110 | 0.223 |
| Nicaragua | 0.312 | 0.095 | -0.248 | -0.049 | -0.050 | -0.011 | -0.024 |
| Argentina | -0.342 | -0.023 | -0.135 | -0.172 | -0.106 | 0.000 | -0.403 |
| Brazil | -0.169 | 0.085 | -0.025 | 0.013 | -0.070 | 0.009 | 0.111 |
| Colombia | 0.012 | 0.093 | -0.154 | 0.111 | -0.067 | 0.026 | -0.080 |
| Costa Rica | -0.455 | 0.024 | 0.009 | -0.240 | -0.072 | -0.001 | -0.090 |
| Dominican Republic | -0.546 | -0.059 | -0.153 | 0.099 | -0.104 | -0.006 | -0.854 |
| Ecuador | -0.236 | 0.092 | 0.006 | 0.133 | -0.071 | -0.035 | 0.148 |
| Guatemala | 0.223 | 0.138 | -0.186 | 0.062 | -0.087 | -0.090 | -0.116 |
| Guyana | 0.237 | 0.091 | -0.116 | 0.139 | 0.019 | 0.004 | 0.010 |
| Jamaica | 0.260 | 0.095 | -0.124 | 0.134 | -0.057 | 0.048 | 0.171 |
| Mexico | 0.318 | 0.118 | -0.148 | -0.009 | 0.023 | 0.018 | 0.289 |
| Panama | -0.309 | 0.065 | -0.039 | 0.044 | -0.107 | 0.012 | -0.389 |
| Paraguay | -0.095 | 0.083 | -0.061 | 0.124 | 0.017 | -0.016 | 0.432 |
| Peru | -0.333 | 0.166 | 0.026 | 0.120 | -0.039 | 0.008 | 0.069 |
| Suriname | 0.210 | 0.081 | -0.038 | 0.147 | -0.079 | 0.042 | 0.111 |
| Venezuela | 0.209 | -0.007 | -0.099 | 0.044 | -0.147 | 0.003 | -0.099 |
| **High income** |  |  |  |  |  |  |  |
| Bahamas | 0.249 | 0.106 | -0.145 | 0.149 | -0.073 | 0.038 | 0.119 |
| Barbados | 0.143 | 0.046 | -0.263 | 0.090 | -0.100 | -0.009 | -1.141 |
| Canada | -0.501 | -0.411 | -0.125 | -0.077 | -0.003 | 0.164 | -0.177 |
| Chile | -0.714 | 0.048 | -0.086 | 0.062 | -0.035 | 0.040 | 0.488 |
| Trinidad and Tobago | -0.055 | 0.082 | -0.062 | 0.146 | 0.312 | 0.078 | 0.191 |
| United States | -0.605 | -0.419 | -0.093 | 0.075 | 0.149 | 0.109 | -0.093 |
| Uruguay | -0.805 | -0.015 | -0.089 | -0.106 | -0.031 | 0.033 | -0.127 |
| **Eastern Mediterranean** |  |  |  |  |  |  |  |
| Afghanistan | 0.378 | 0.107 | -0.127 | 0.131 | 0.195 | -0.084 | 0.388 |
| Somalia | 0.102 | -0.200 | -0.818 | -0.398 | -0.178 | -0.148 | -0.962 |
| Syria | 0.379 | 0.102 | -0.233 | 0.181 | 0.360 | 0.120 | 0.717 |
| Djibouti | 0.194 | 0.034 | -0.651 | -0.375 | -0.530 | -0.121 | -4.140 |
| Egypt | 0.238 | 0.041 | -0.122 | 0.153 | 0.927 | -0.005 | -0.294 |
| Iran | -0.300 | 0.053 | -0.014 | 0.093 | 0.189 | -0.031 | -0.013 |
| Morocco | -0.301 | 0.053 | -0.108 | 0.103 | 0.175 | 0.008 | -0.147 |
| Pakistan | 0.227 | 0.085 | -0.151 | 0.124 | -0.101 | -0.075 | -0.234 |
| Tunisia | -0.261 | 0.070 | -0.067 | 0.109 | 0.064 | 0.031 | 0.287 |
| Iraq | 0.308 | 0.067 | -0.084 | 0.102 | 0.120 | -0.111 | -0.192 |
| Jordan | 0.226 | 0.060 | -0.165 | 0.044 | 0.729 | -0.170 | -1.354 |
| Lebanon | 0.093 | 0.036 | -0.189 | -0.029 | -0.038 | 0.032 | -0.603 |
| Libya | 0.256 | 0.026 | 0.025 | 0.122 | 0.172 | -0.091 | -0.331 |
| **High income** |  |  |  |  |  |  |  |
| Bahrain | -1.015 | -0.958 | -0.392 | -0.036 | 0.366 | -0.541 | -0.862 |
| Kuwait | -0.357 | -0.723 | -0.039 | 0.054 | 0.367 | -0.440 | -1.503 |
| Oman | 0.132 | -0.461 | -0.151 | 0.088 | 0.301 | -0.304 | -0.719 |
| Qatar | -0.236 | -0.005 | -0.235 | -0.041 | -0.124 | -0.501 | -1.439 |
| Saudi Arabia | -0.687 | -0.178 | -0.250 | -0.131 | 0.352 | -0.258 | -0.554 |
| United Arab Emirates | -0.482 | -0.752 | -0.246 | 0.017 | 0.349 | -0.433 | -0.646 |
| **Europe** |  |  |  |  |  |  |  |
| Kyrgyzstan | 0.303 | 0.105 | -0.199 | 0.130 | -0.024 | -0.099 | 0.171 |
| Ukraine | 0.213 | 0.074 | -0.101 | 0.123 | 0.187 | 0.105 | 0.314 |
| Uzbekistan | 0.286 | 0.170 | -0.023 | 0.165 | 0.131 | -0.109 | -0.152 |
| Albania | -0.189 | 0.145 | -0.148 | 0.005 | -0.007 | 0.034 | -0.530 |
| Azerbaijan | -0.204 | 0.132 | -0.037 | 0.105 | 0.146 | 0.002 | 0.180 |
| Belarus | 0.195 | -0.030 | -0.103 | 0.099 | -0.074 | 0.027 | -0.063 |
| Bosnia and Herzegovina | 0.173 | 0.058 | -0.073 | 0.109 | 0.270 | 0.178 | 0.311 |
| Bulgaria | 0.229 | 0.027 | -0.136 | 0.098 | 0.245 | 0.138 | 0.303 |
| Georgia | 0.211 | 0.046 | -0.136 | 0.115 | 0.021 | 0.030 | -0.255 |
| Kazakhstan | -0.074 | 0.021 | -0.067 | 0.100 | -0.031 | 0.021 | 0.067 |
| Moldova | 0.221 | 0.058 | -0.144 | 0.118 | -0.025 | 0.041 | 0.130 |
| Romania | 0.183 | 0.053 | -0.246 | -0.012 | 0.050 | 0.131 | 0.196 |
| Russia | 0.064 | 0.041 | -0.061 | 0.111 | 0.255 | 0.066 | 0.324 |
| Serbia | -0.600 | 0.171 | -0.231 | 0.097 | 0.230 | 0.126 | 0.255 |
| Turkey | -0.916 | 0.068 | -0.157 | 0.057 | -0.128 | -0.033 | 0.122 |
| **High income** |  |  |  |  |  |  |  |
| Andorra | -0.285 | -0.154 | -0.175 | -0.412 | -0.162 | 0.031 | -1.137 |
| Austria | -0.735 | -0.474 | -0.267 | -0.088 | -0.035 | 0.184 | -0.061 |
| Belgium | -0.673 | -0.555 | -0.066 | -0.084 | -0.060 | 0.153 | -0.278 |
| Croatia | -0.192 | 0.030 | -0.072 | 0.087 | -0.010 | 0.133 | -0.070 |
| Cyprus | -0.648 | -0.707 | -0.146 | -0.094 | -0.031 | -0.155 | -0.187 |
| Czechia | -0.658 | -0.550 | -0.115 | 0.040 | 0.138 | 0.175 | 0.126 |
| Denmark | -0.693 | -0.574 | -0.200 | -0.115 | -0.063 | 0.108 | -0.926 |
| Estonia | -0.663 | -0.460 | -0.113 | 0.075 | 0.094 | 0.138 | -0.366 |
| Finland | -0.704 | -0.354 | 0.018 | -0.433 | -0.110 | 0.133 | -0.284 |
| France | -0.844 | 0.008 | -0.146 | -0.639 | -0.104 | 0.141 | -0.493 |
| Germany | -0.699 | -0.473 | -0.061 | -0.099 | -0.032 | 0.126 | 0.157 |
| Greece | -0.628 | -0.023 | -0.121 | -0.044 | -0.094 | 0.103 | 0.074 |
| Hungary | -0.581 | 0.093 | -0.197 | 0.105 | 0.267 | 0.134 | 0.503 |
| Iceland | -0.976 | -0.573 | -0.221 | -0.472 | -0.064 | -0.135 | -0.765 |
| Ireland | -0.806 | -0.771 | -0.087 | -0.082 | -0.060 | -0.080 | -0.400 |
| Israel | -0.737 | 0.129 | -0.363 | -0.094 | -0.168 | -0.180 | -1.138 |
| Italy | -0.777 | -0.601 | -0.089 | -0.106 | 0.045 | 0.158 | -0.073 |
| Latvia | -0.253 | 0.014 | -0.007 | 0.093 | -0.077 | 0.132 | -0.080 |
| Lithuania | -0.696 | -0.310 | -0.114 | 0.082 | 0.046 | 0.157 | 0.057 |
| Luxembourg | -0.760 | -0.781 | -0.068 | -0.090 | -0.060 | -0.039 | -0.352 |
| Malta | -0.645 | 0.419 | -0.108 | -0.072 | -0.167 | 0.177 | -0.981 |
| Monaco | -0.261 | -0.142 | -0.221 | -0.129 | -0.188 | 0.019 | -0.510 |
| Netherlands | -0.776 | -0.881 | -0.094 | -0.118 | -0.078 | 0.116 | -1.036 |
| Norway | -0.799 | -0.498 | -0.078 | -0.520 | -0.111 | 0.111 | -0.637 |
| Poland | -0.039 | 0.040 | -0.041 | 0.083 | -0.051 | 0.160 | 0.458 |
| Portugal | -0.702 | -0.036 | 0.031 | -0.262 | -0.110 | 0.103 | -0.445 |
| Slovakia | -0.861 | -0.468 | -0.155 | 0.082 | -0.010 | 0.004 | -0.018 |
| Slovenia | -0.785 | -0.555 | -0.079 | 0.031 | -0.034 | 0.176 | -0.051 |
| Spain | -0.675 | -0.197 | -0.144 | -0.505 | -0.098 | 0.160 | -0.321 |
| Sweden | -0.601 | -0.514 | -0.091 | -0.083 | -0.088 | 0.128 | -0.530 |
| Switzerland | -0.732 | -0.819 | -0.264 | -0.087 | -0.084 | 0.127 | -0.844 |
| United Kingdom | -0.852 | -0.176 | -0.150 | -0.441 | -0.095 | 0.166 | -0.332 |
| **South-East Asia** |  |  |  |  |  |  |  |
| Bangladesh | 0.165 | 0.072 | -0.150 | -0.185 | -0.048 | -0.061 | -0.176 |
| India | 0.292 | 0.164 | -0.465 | 0.153 | -0.065 | 0.056 | -0.240 |
| Indonesia | 0.295 | 0.069 | -0.088 | 0.117 | -0.076 | 0.002 | 0.216 |
| Myanmar | 0.154 | 0.070 | -0.073 | 0.093 | -0.047 | -0.017 | -0.561 |
| Nepal | 0.120 | 0.190 | -0.192 | -0.012 | -0.096 | -0.010 | -0.693 |
| Sri Lanka | -0.083 | 0.162 | 0.106 | 0.064 | -0.052 | 0.006 | 0.497 |
| Timor | 0.120 | 0.178 | -0.617 | -0.037 | -0.153 | -0.372 | -2.355 |
| Thailand | -0.472 | 0.022 | 0.001 | -0.247 | -0.054 | -0.004 | -0.319 |
| **Western Pacific** |  |  |  |  |  |  |  |
| Cambodia | -2.601 | 0.025 | -0.044 | 0.086 | -0.147 | -0.265 | -1.641 |
| Mongolia | -0.504 | 0.050 | -0.102 | 0.106 | -0.008 | -0.187 | -0.755 |
| Papua New Guinea | 0.276 | 0.080 | 0.296 | 0.062 | -0.089 | -0.109 | -0.415 |
| Philippines | 0.223 | 0.203 | -0.036 | 0.045 | -0.006 | -0.074 | 0.184 |
| Vietnam | -0.288 | 0.160 | -0.054 | 0.087 | -0.073 | 0.027 | -0.010 |
| China | -0.337 | -0.025 | -0.130 | 0.075 | -0.092 | 0.042 | -8.508 |
| Fiji | -0.027 | 0.051 | 0.058 | 0.072 | -0.028 | -0.052 | -0.185 |
| Malaysia | -0.548 | 0.019 | -0.030 | 0.054 | -0.078 | -0.132 | 0.051 |
| **High income** |  |  |  |  |  |  |  |
| Australia | -0.182 | -0.655 | -0.080 | -0.134 | -0.072 | 0.046 | -1.056 |
| Brunei | -0.563 | -2.368 | -3.355 | 0.078 | -0.070 | -0.341 | -2.627 |
| Japan | 0.192 | -0.574 | -0.046 | 0.071 | -0.076 | 0.107 | -0.555 |
| New Zealand | -0.277 | -0.720 | -0.055 | -0.124 | -0.063 | -0.124 | -1.116 |
| Singapore | -0.734 | 0.101 | 0.005 | 0.082 | -0.140 | -0.064 | -1.097 |
| South Korea | -0.637 | -0.424 | 0.089 | 0.079 | -0.021 | 0.001 | 0.386 |

## 2.5 Change rate of CFR after simulating a 5% increase in vaccination for each country

| **Driving factor** | **Entity** | **Change rate of CFR** |
| --- | --- | --- |
| **Class 1: low vaccination coverage** | Niger | 0.818 |
|  | Syria | 0.631 |
|  | Tanzania | 0.615 |
|  | Sudan | 0.603 |
|  | Afghanistan | 0.603 |
|  | Mexico | 0.573 |
|  | Nicaragua | 0.539 |
|  | Uganda | 0.539 |
|  | Honduras | 0.532 |
|  | Cameroon | 0.529 |
|  | Bahamas | 0.514 |
|  | Papua New Guinea | 0.491 |
|  | Suriname | 0.477 |
|  | Iraq | 0.473 |
|  | Guyana | 0.462 |
|  | Namibia | 0.446 |
|  | Senegal | 0.445 |
|  | Indonesia | 0.437 |
|  | Libya | 0.434 |
|  | Haiti | 0.429 |
|  | Ghana | 0.416 |
|  | Angola | 0.414 |
|  | Guinea | 0.413 |
|  | Burkina Faso | 0.395 |
|  | Kyrgyzstan | 0.377 |
|  | South Africa | 0.367 |
|  | Congo | 0.364 |
|  | Zimbabwe | 0.361 |
|  | Moldova | 0.360 |
|  | Algeria | 0.359 |
|  | Mali | 0.354 |
|  | Georgia | 0.348 |
|  | Ukraine | 0.348 |
|  | Jamaica | 0.339 |
|  | Chad | 0.312 |
|  | Guatemala | 0.311 |
|  | Uzbekistan | 0.300 |
|  | Romania | 0.287 |
|  | Philippines | 0.282 |
|  | Venezuela | 0.276 |
|  | Belarus | 0.270 |
|  | Cote d'Ivoire | 0.237 |
|  | Cape Verde | 0.233 |
|  | Sierra Leone | 0.210 |
|  | Malawi | 0.200 |
|  | Togo | 0.192 |
|  | Nigeria | 0.187 |
|  | Barbados | 0.187 |
|  | India | 0.186 |
|  | Gabon | 0.168 |
|  | Lesotho | 0.138 |
|  | Liberia | 0.132 |
|  | Japan | 0.097 |
|  | Burundi | 0.000 |
|  | Benin | 0.000 |
|  | Bangladesh | 0.000 |
|  | Botswana | 0.000 |
|  | Central African Republic | 0.000 |
|  | Democratic Republic of Congo | 0.000 |
|  | Djibouti | 0.000 |
|  | Ethiopia | 0.000 |
|  | Gambia | 0.000 |
|  | Kenya | 0.000 |
|  | Lebanon | 0.000 |
|  | Myanmar | 0.000 |
|  | Mozambique | 0.000 |
|  | Pakistan | 0.000 |
|  | Rwanda | 0.000 |
|  | Somalia | 0.000 |
|  | Zambia | 0.000 |
| **Class 2: Aging** | Austria | 0.272 |
|  | Uruguay | 0.268 |
|  | Estonia | 0.237 |
|  | Lithuania | 0.161 |
|  | Czechia | 0.134 |
|  | Slovenia | 0.119 |
|  | Denmark | 0.119 |
|  | Netherlands | 0.117 |
|  | Switzerland | 0.068 |
|  | Latvia | 0.064 |
|  | Norway | 0.043 |
|  | Germany | 0.035 |
|  | Sweden | 0.033 |
|  | Andorra | 0.028 |
|  | Croatia | 0.017 |
|  | Spain | 0.011 |
|  | Monaco | 0.004 |
|  | Australia | 0.000 |
|  | Belgium | 0.000 |
|  | Canada | 0.000 |
|  | Finland | 0.000 |
|  | France | 0.000 |
|  | United Kingdom | 0.000 |
|  | Greece | 0.000 |
|  | Italy | 0.000 |
|  | Portugal | 0.000 |
| **Class 3: high disease burden** | Mongolia | 0.796 |
|  | Bulgaria | 0.515 |
|  | El Salvador | 0.495 |
|  | Bosnia and Herzegovina | 0.445 |
|  | Egypt | 0.428 |
|  | Jordan | 0.414 |
|  | Brunei | 0.406 |
|  | Russia | 0.398 |
|  | Trinidad and Tobago | 0.336 |
|  | China | 0.312 |
|  | Belize | 0.282 |
|  | Hungary | 0.261 |
|  | Malaysia | 0.248 |
|  | Colombia | 0.190 |
|  | United Arab Emirates | 0.189 |
|  | Kazakhstan | 0.161 |
|  | Azerbaijan | 0.139 |
|  | Serbia | 0.115 |
|  | Slovakia | 0.077 |
|  | Ecuador | 0.071 |
|  | Dominican Republic | 0.064 |
|  | Morocco | 0.029 |
|  | Fiji | 0.028 |
|  | Iran | 0.023 |
|  | Saudi Arabia | 0.022 |
|  | Tunisia | 0.018 |
|  | Bahrain | 0.017 |
|  | Kuwait | 0.012 |
|  | United States | 0.012 |
|  | Madagascar | 0.006 |
|  | Cambodia | 0.000 |
|  | Oman | 0.000 |
| **Class 4: low GDP per capita** | Vietnam | 0.795 |
|  | Bolivia | 0.243 |
|  | Peru | 0.162 |
|  | Albania | 0.155 |
|  | Mauritius | 0.092 |
|  | Seychelles | 0.066 |
|  | Panama | 0.052 |
|  | Thailand | 0.043 |
|  | Costa Rica | 0.017 |
|  | Israel | 0.000 |
|  | Malta | 0.000 |
|  | Nepal | 0.000 |
|  | Singapore | 0.000 |
|  | Timor | 0.000 |
| **Class 5: Other** | Sri Lanka | 0.952 |
|  | Poland | 0.647 |
|  | Chile | 0.252 |
|  | Ireland | 0.164 |
|  | Luxembourg | 0.162 |
|  | Paraguay | 0.065 |
|  | Brazil | 0.053 |
|  | New Zealand | 0.009 |
|  | Argentina | 0.000 |
|  | Cyprus | 0.000 |
|  | Iceland | 0.000 |
|  | South Korea | 0.000 |
|  | Qatar | 0.000 |
|  | Turkey | 0.000 |

# Section 3: Figures

## 3.1 Cross-correlation analysis for COVID-19 deaths and cases


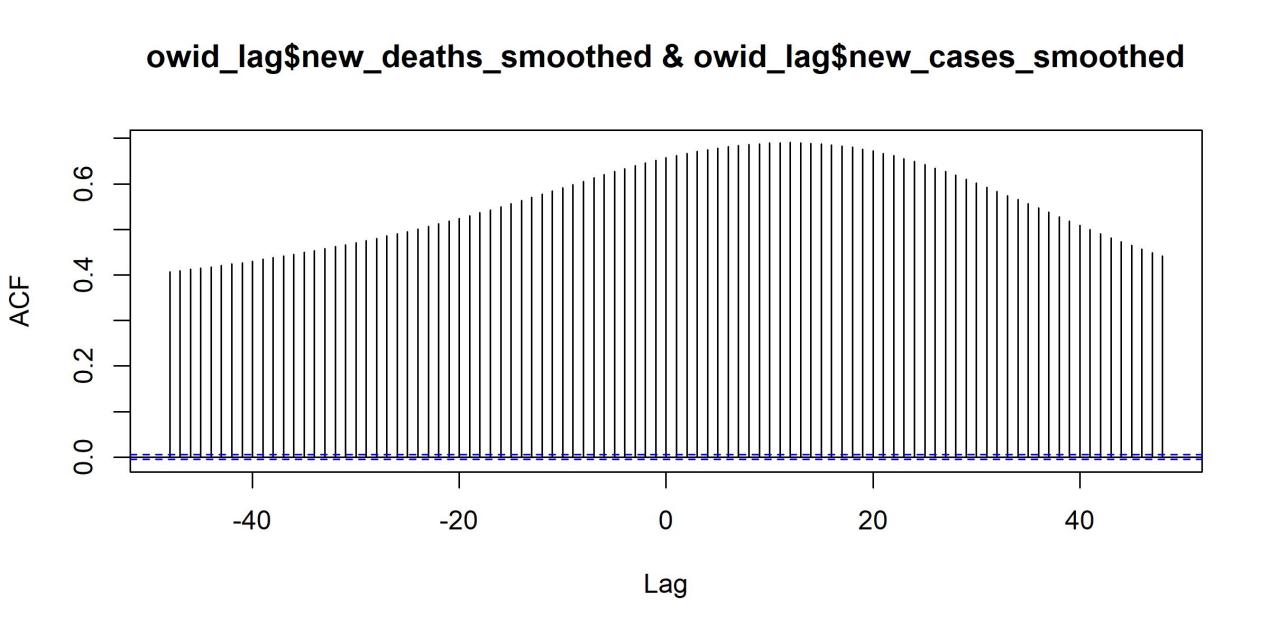


## 3.2 Correlation between covariates


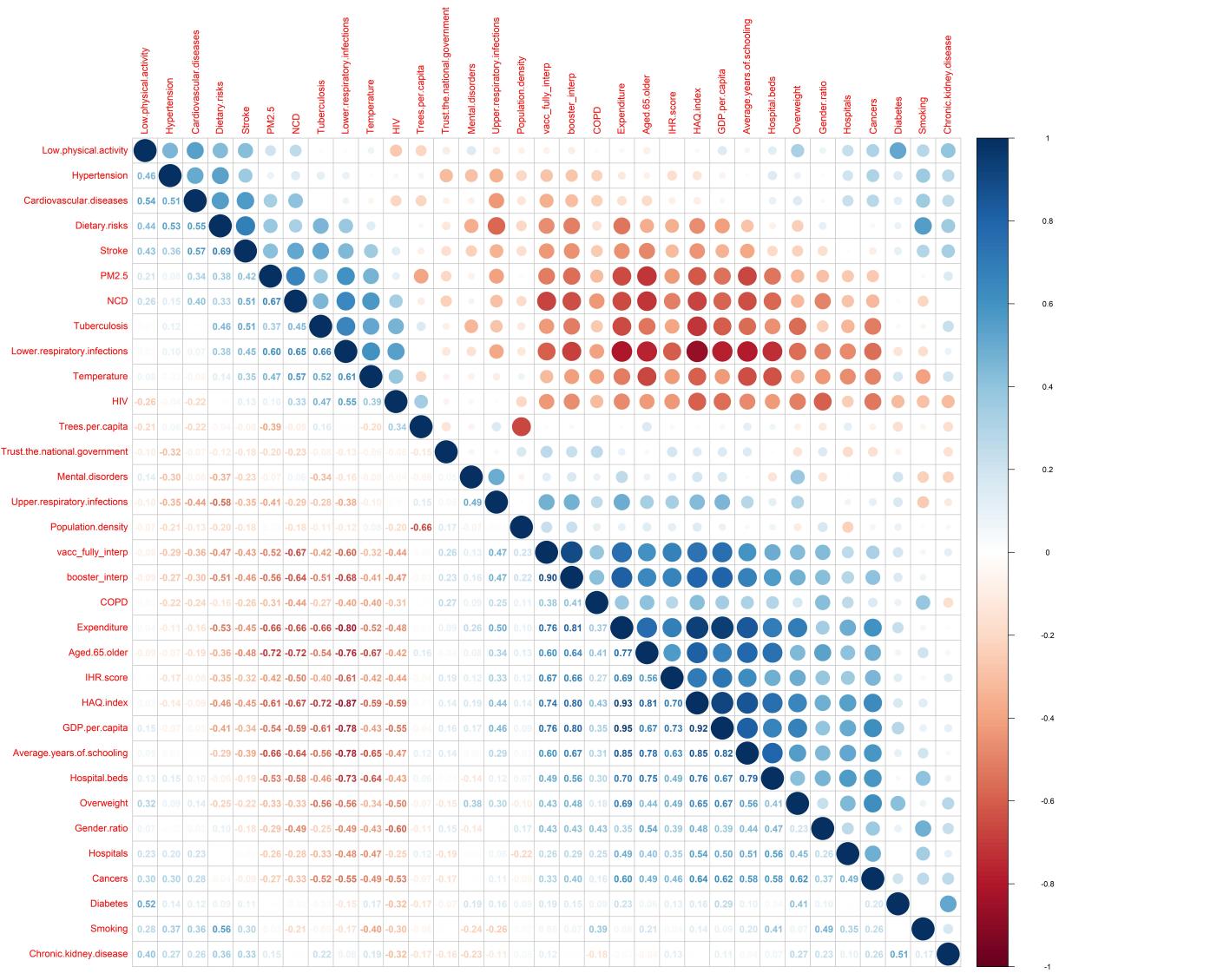


## 3.3 Univariate correlation between CFRs and vaccination rate and HAQ index


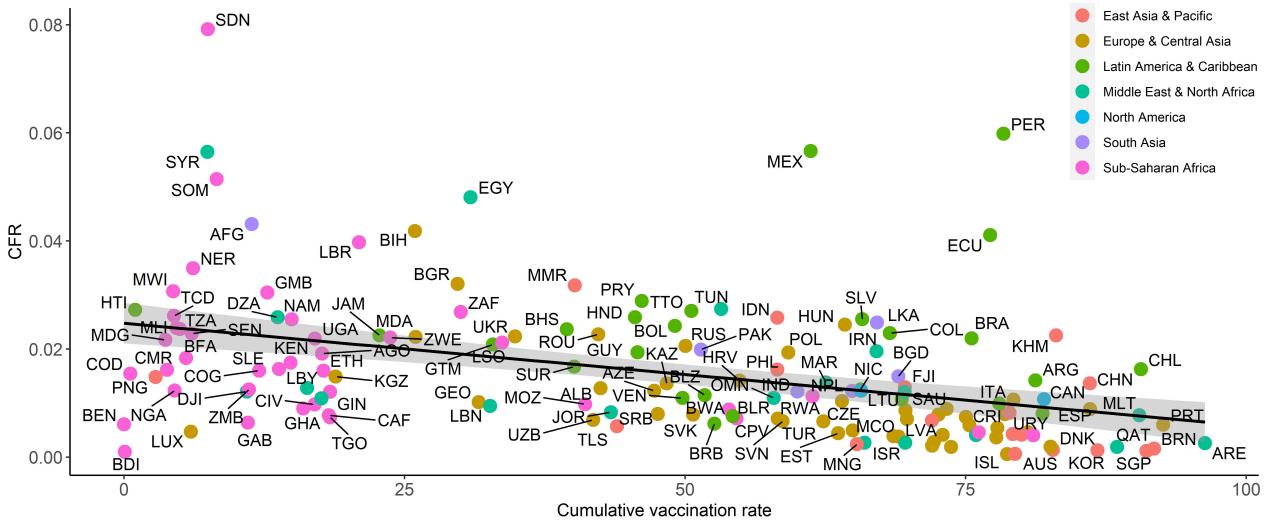


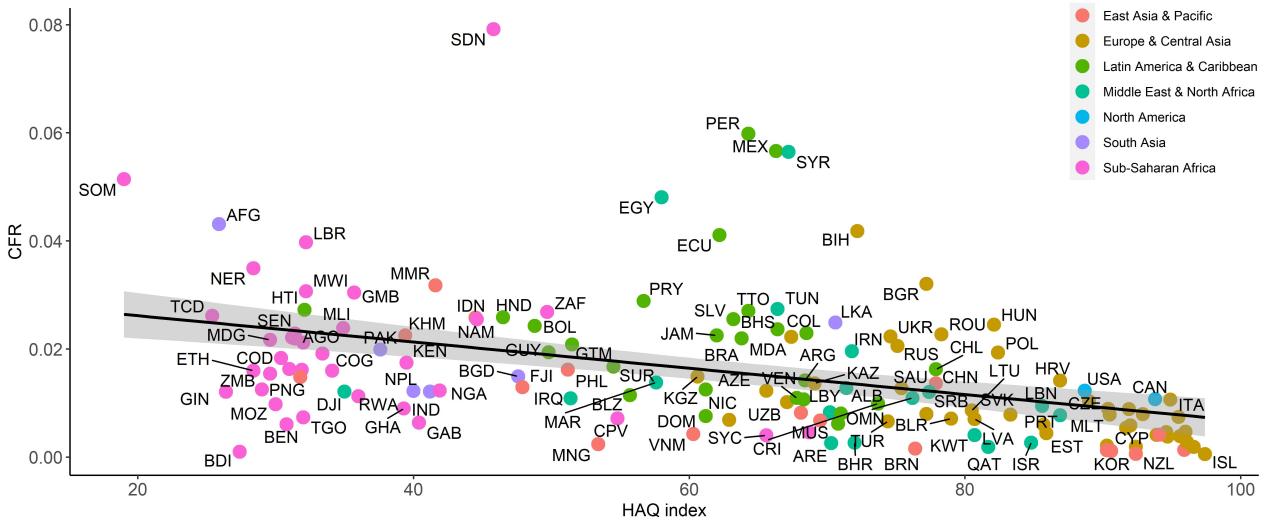


## 3.4 CFRs for countries in four income levels by period


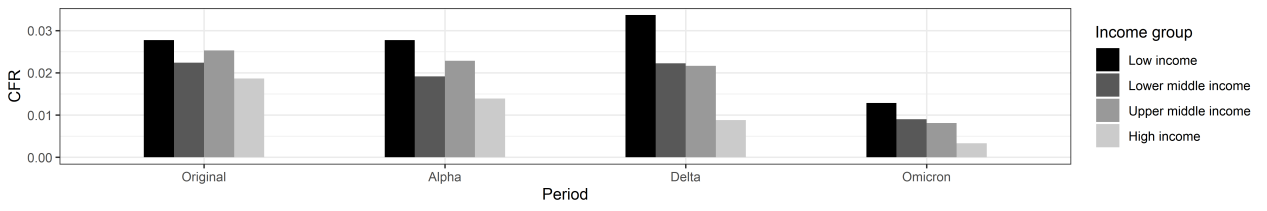


## 3.5 SHAP dependence plot for each covariate in four period model


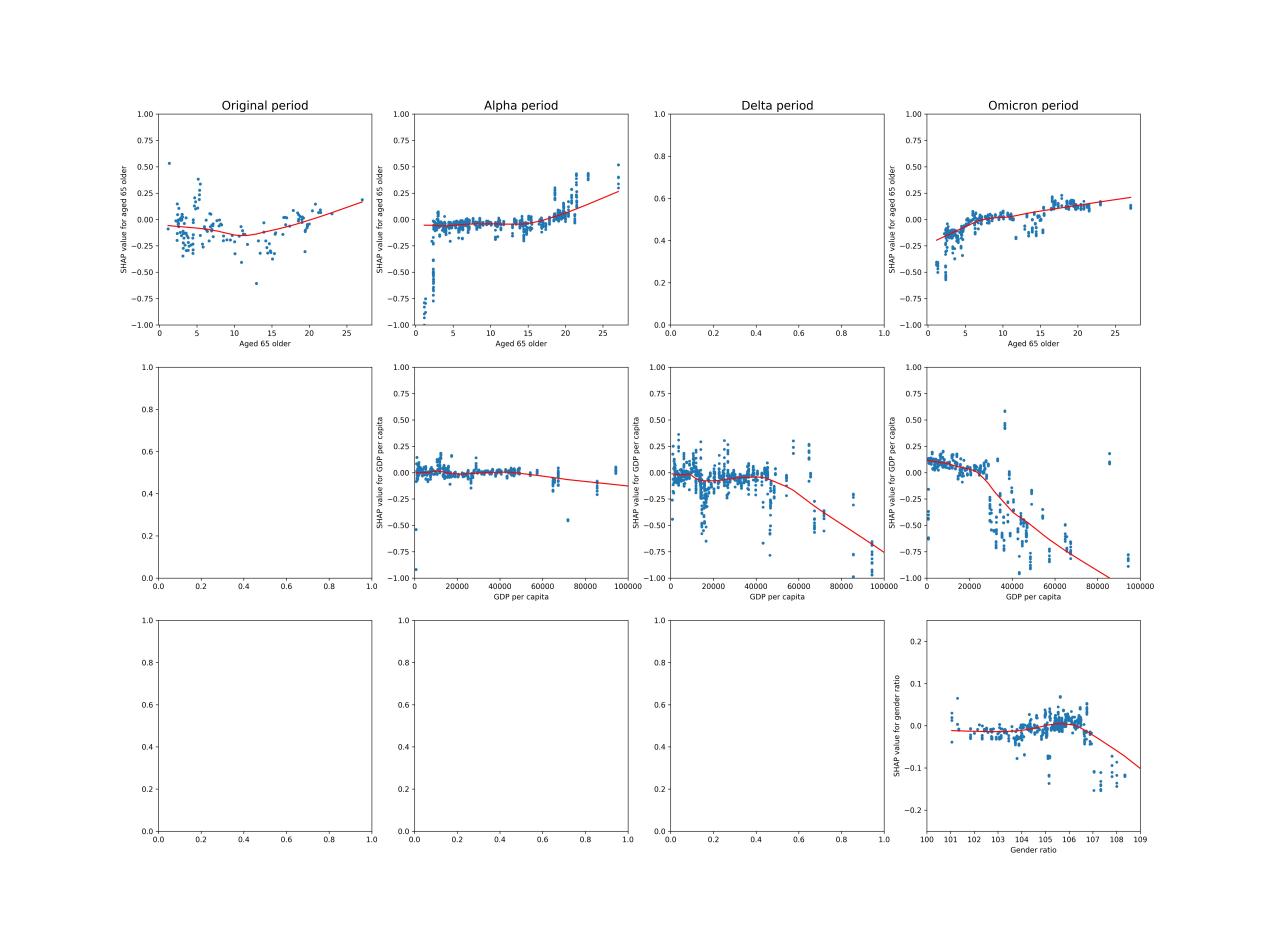


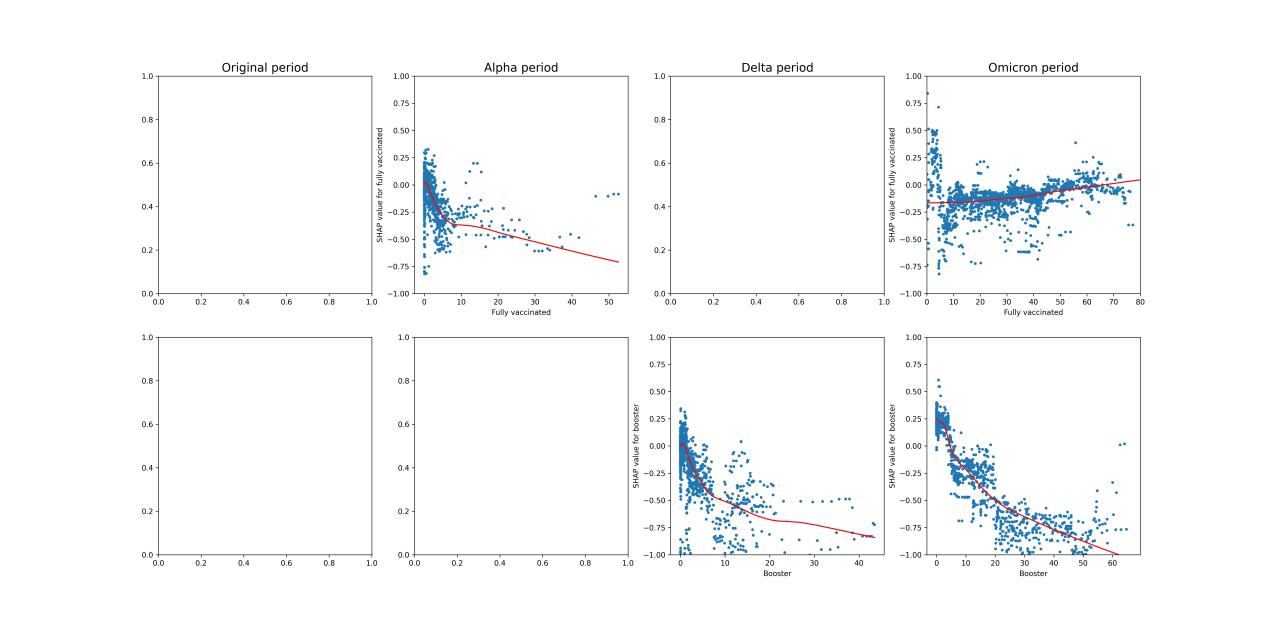


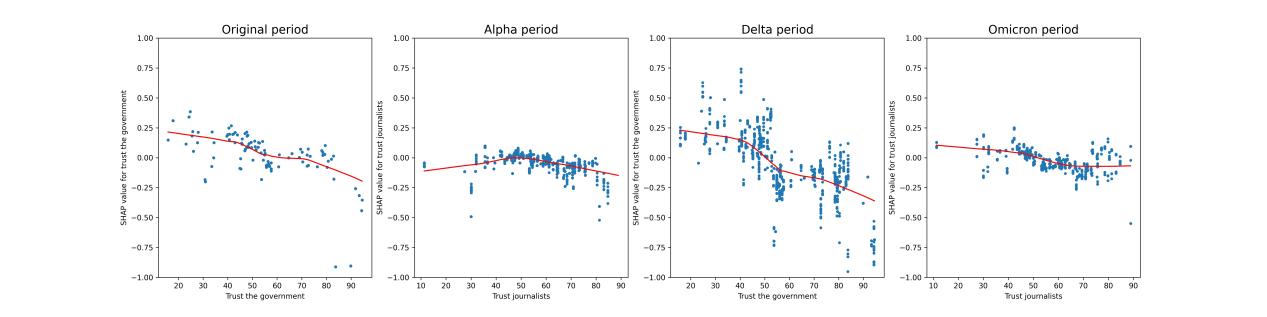


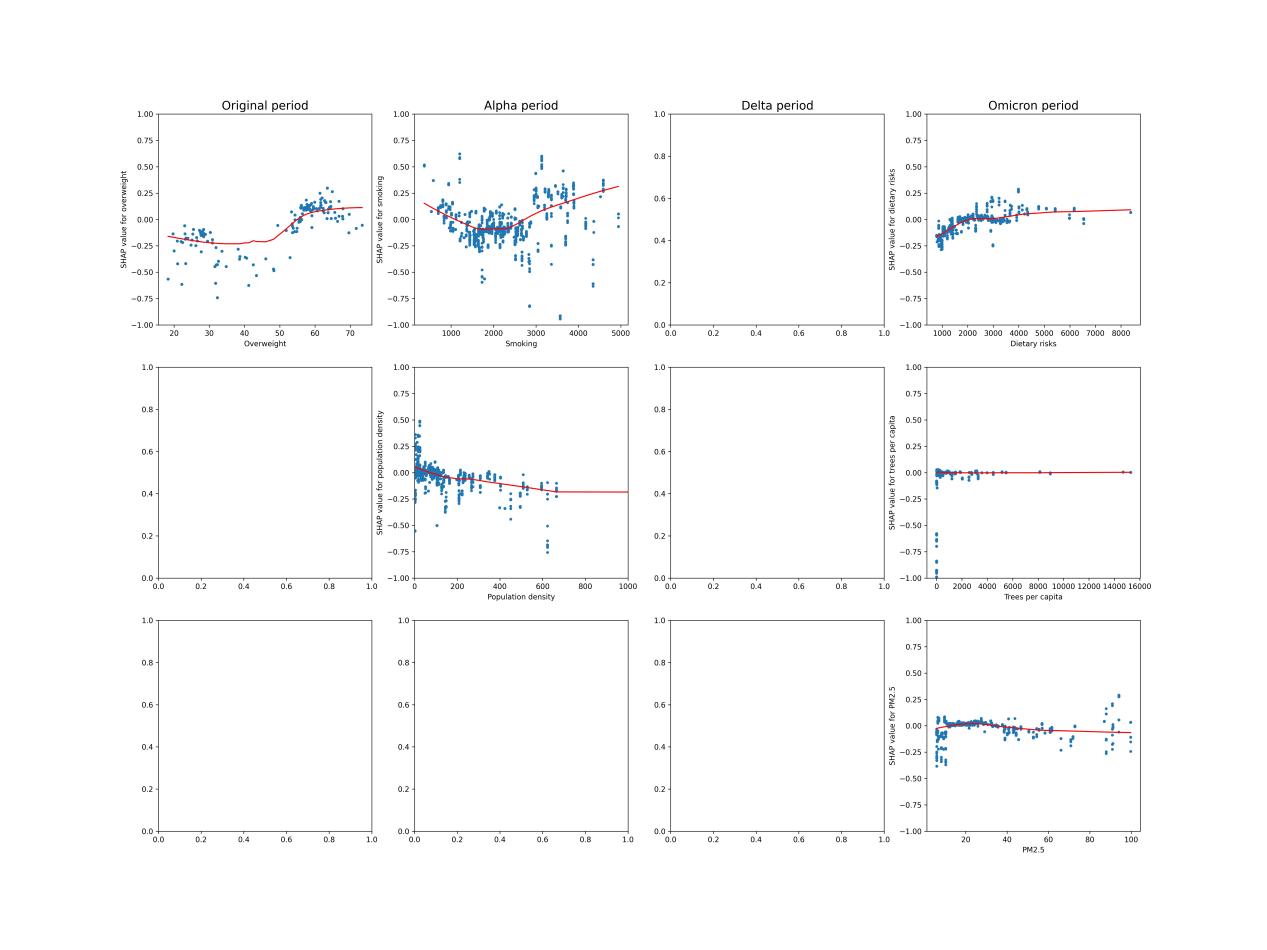


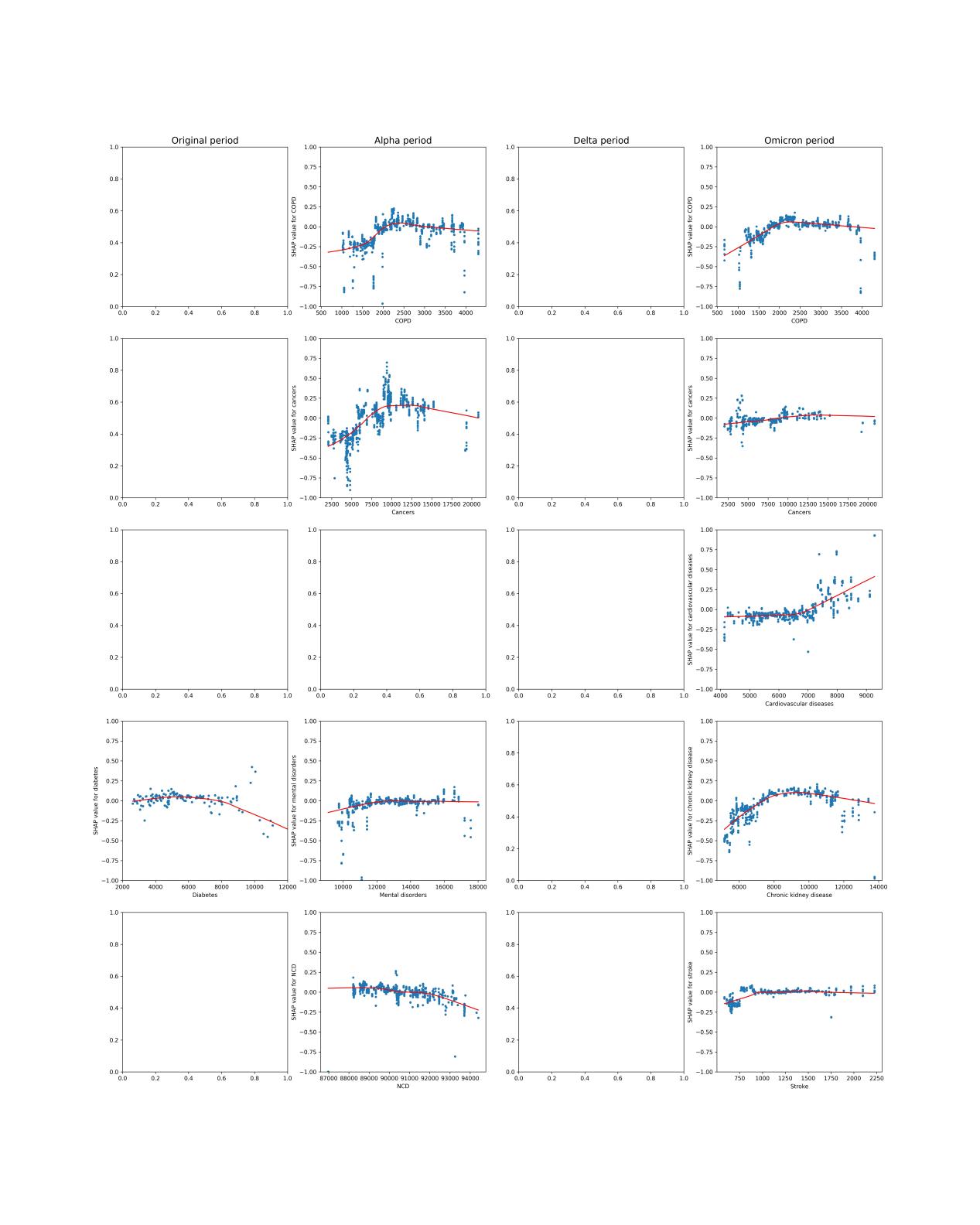


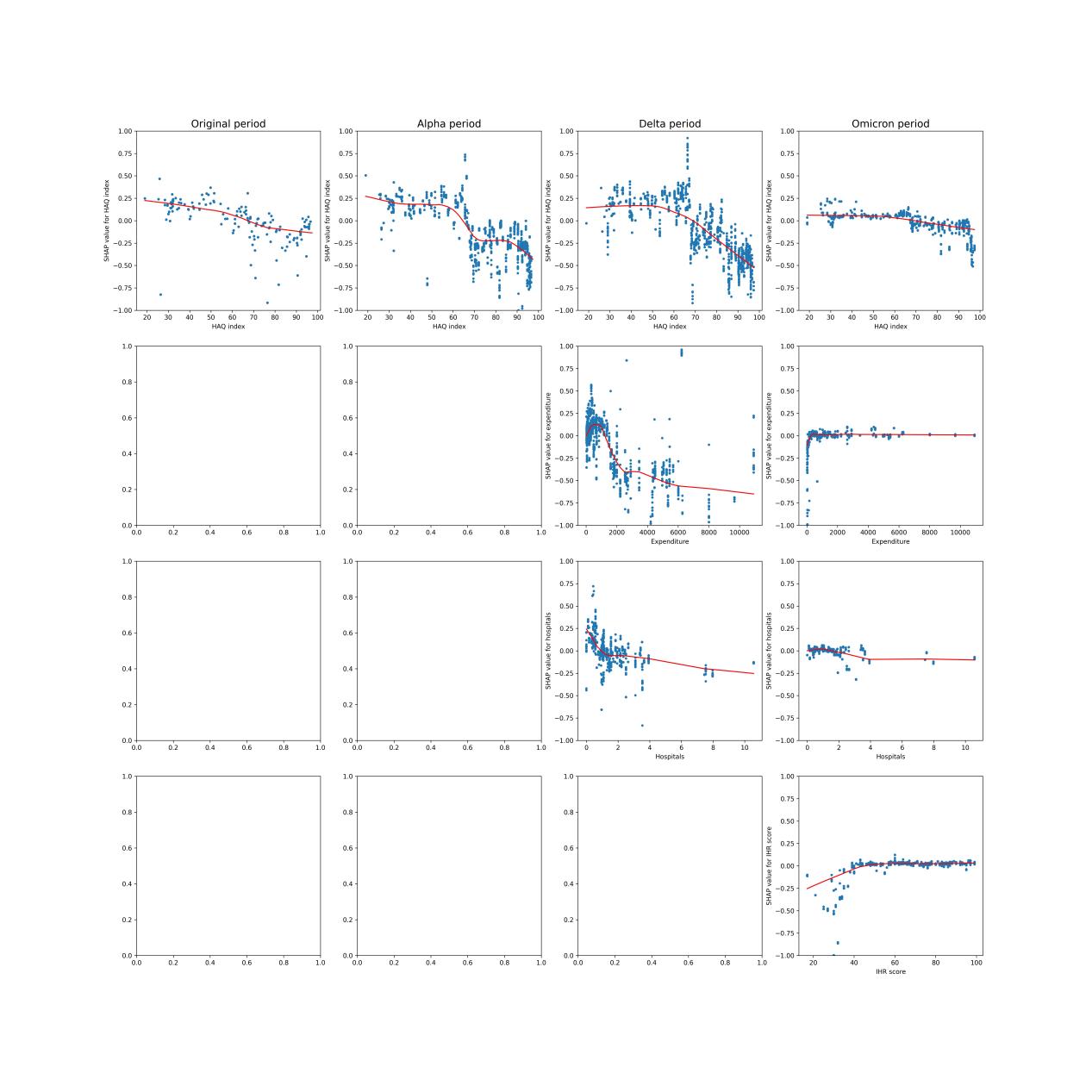


## 3.6 Distribution and cross-Class differences in the change rate of CFR after simulating a 5%, 10%, 15%, 20% increase in vaccination.


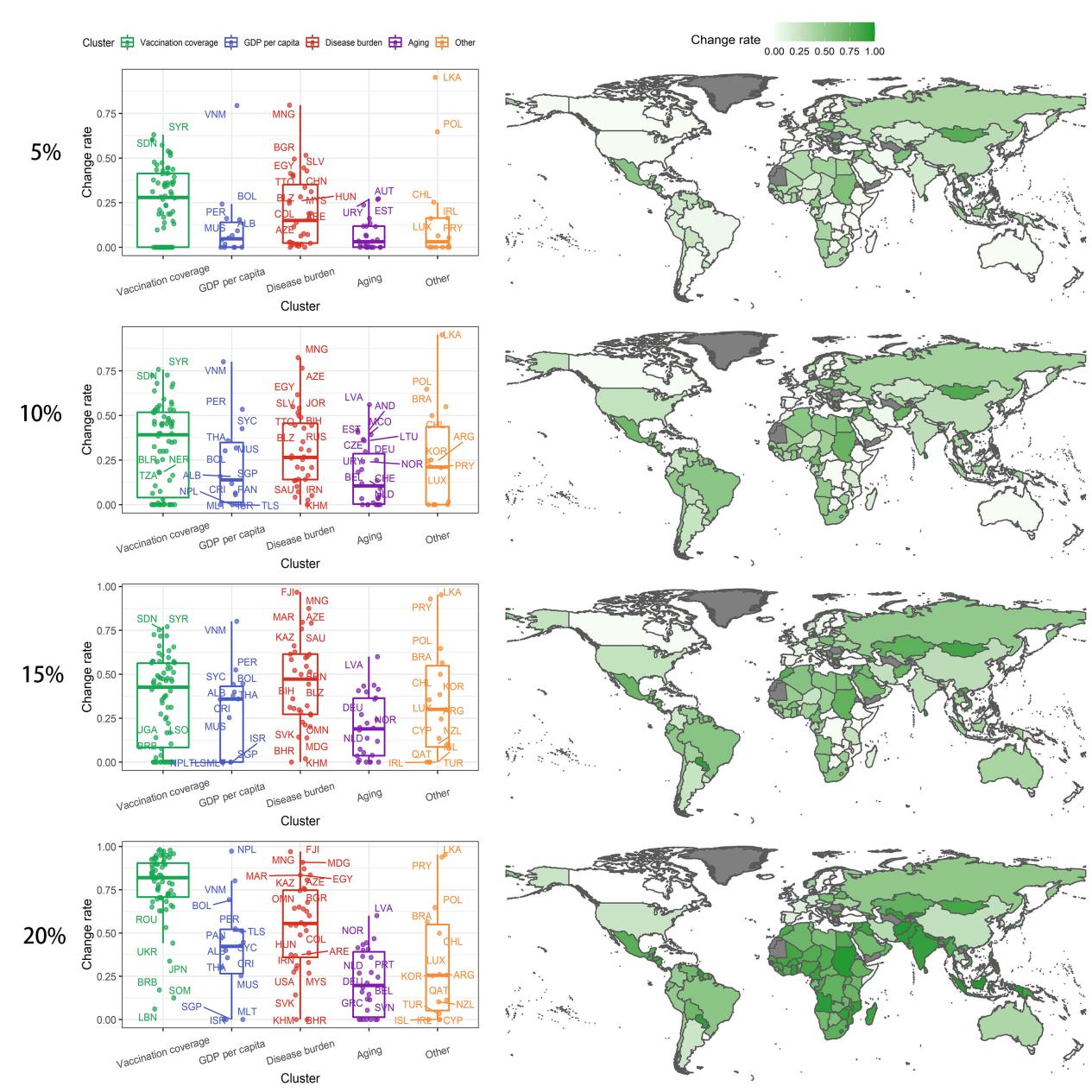

Supplement: Supplementary file 1 — Supplementary material 1. [file 12963_2024_330_MOESM1_ESM.docx]
